# Neural signatures of imaginary motivational states: desire for music, movement and social play

**DOI:** 10.1007/s10548-024-01047-1

**Published:** 2024-04-16

**Authors:** Giada Della Vedova, Alice Mado Proverbio

**Affiliations:** 1 Cognitive Electrophysiology lab, Dept. of Psychology, University of Milano, Bicocca, Italy; 2 NeuroMI, Milan Center for Neuroscience, Milan, Italy; 3 Department of Psychology of University of Milano-Bicocca, Piazza dell’Ateneo nuovo 1, Milan, 20162 Italy

**Keywords:** Imagery, Event-related potentials (ERPs), Craving, Motivation, Emotion, BCI

## Abstract

The literature has demonstrated the potential for detecting accurate electrical signals that correspond to the will or intention to move, as well as decoding the thoughts of individuals who imagine houses, faces or objects. This investigation examines the presence of precise neural markers of imagined motivational states through the combining of electrophysiological and neuroimaging methods. 20 participants were instructed to vividly imagine the desire to move, listen to music or engage in social activities. Their EEG was recorded from 128 scalp sites and analysed using individual standardized Low-Resolution Brain Electromagnetic Tomographies (LORETAs) in the N400 time window (400–600 ms). The activation of 1056 voxels was examined in relation to the 3 motivational states. The most active dipoles were grouped in eight regions of interest (ROI), including Occipital, Temporal, Fusiform, Premotor, Frontal, OBF/IF, Parietal, and Limbic areas. The statistical analysis revealed that all motivational imaginary states engaged the right hemisphere more than the left hemisphere. Distinct markers were identified for the three motivational states. Specifically, the right temporal area was more relevant for “Social Play”, the orbitofrontal/inferior frontal cortex for listening to music, and the left premotor cortex for the “Movement” desire. This outcome is encouraging in terms of the potential use of neural indicators in the realm of brain-computer interface, for interpreting the thoughts and desires of individuals with locked-in syndrome.

**Supplementary Information:**

The online version contains supplementary material available at 10.1007/s10548-024-01047-1.

## Introduction

The objective of this research was to pinpoint neural indicators for imaginative and motivational processes, using electromagnetic signals such as the N400 component of ERPs. The potential for detecting, at least in broad classes, the mental content of perfectly still and silent individuals was explored here by using a neuroimaging technique and subjecting the results of individual source reconstructions to statistical analysis to identify the brain region whose activation was most characteristic of a particular mental state. This study aimed to develop a method suitable for Brain Computer Interface (BCI) purposes, in order to detect, at least roughly, the patients’ physiological needs and desires. In Proverbio and Pischedda (2023a) it was shown how secondary motivational states (such as, desire for music, movement or social play) can be detected through ERPs, but literature on non-compelling urges is very scarce. Extensive research within the addiction field has described ‘craving’ has an exceedingly compelling but subjective state that individuals struggle to resist. In contrast, desire denotes a less intense level of wanting (Peterson-Sockwell et al. 2023). While biological determinants of craving have been extensively examined (e.g. Antons et al. 2023; Betts et al. 2021; Ferguson and Shiffman 2009), little is known about ‘non-pathological’ motivational states, including desires, urges, and related constructs. These states can vary in strength, specificity, awareness, and intensity (Stults-Kolehmainen et al. 2020), and may have emotional implications. Generally, a ‘desire’ signifies a conscious state of longing or an urge, wherein an individual’s attention is directed towards achieving pleasure, easing discomfort, fulfilling a requirement, or performing actions associated with these desired results (Kavanagh et al. 2005).

Desire is not merely an emotion, even though it displays an affective quality. It involves the psychological occurrence of mental imagery or verbal ideation of the attractive features of desired items or activities (Salkovskis and Reynolds 1994). The importance of imagery in the experience of desire has been emphasized by Kavanagh et al. (2005), as it emulates the sensory and emotional aspects of desired experiences. While images of desire can bring momentary satisfaction, they also highlight physical and emotional inadequacies. Such imagery intensifies cravings and desires, engaging multiple senses. Kavanagh et al. (2005) proposed that it is not the meaning of the envisioned desire that captivates individuals, but rather the sensory components and emotional response it evokes. Research has shown that imagery tasks are an effective strategy for eliciting desires (Devos et al. 2022), where the intensity of the craving experienced is linked to the vividness of the imagined scenario (Harvey et al. 2005). Kavanagh and colleagues (2005) proposed that specific brain regions are activated when experiencing desires or cravings, similar to those activated during sensory imagery within the same sensory category. Indeed, the intention to initiate a movement leads to the activation of motion-related areas, such as the premotor cortex, or the parietal lobe and cerebellum (Decety et al. 1990, 1994). In the same vein, the desire for music might lead to activation of brain regions associated with music imagery, i.e., the prefrontal cortex, the inferior frontal gyrus, or the superior temporal gyrus (Herholz et al. 2012).

On the other hand, ‘mental imagery’ refers to the subjective experience of representing sensory information without an external stimulus. It involves the retrieval of stored memory to allow the individual to re-experience a version of the original stimulus or to combine previously encountered stimuli (Pearson et al. 2015). It is recognised that there are common areas of activation across all forms of imagery, such as the frontal and parietal regions (McNorgan 2012). These areas support short-term memory processes that are essential for the storage and manipulation of information (Chen et al. 2021; Pearson 2019), while the occipital area facilitates perceptual experience (e.g. Winlove et al. 2018; Dijkstra et al. 2019). Furthermore, the neural pathways responsible for perceiving sensory information within a specific modality are also activated during mental imagery (Kosslyn et al. 2001). Research on activation patterns has backed the idea that imagining specific modalities or recalling information from different modalities leads to heightened activation in the corresponding sensorimotor areas of the brain (McNorgan 2012). For example, multiple lines of evidence converge to suggest that motor imagery (MI) shares similarities with the processes involved in planning and preparing real actions, albeit with the distinction that actual execution is inhibited at some point within the corticospinal pathway (Jeannerod and Decety 1995). Extensive research has been conducted on motor imagery within the field of brain-computer interfaces. For instance, Yuan et al. (2010) investigated brain activity triggered by motor imagery and actual movements through fMRI and EEG source imaging. The utilization of EEG source imaging for studying motor imagery dates back to 2004 (Qin et al. 2004) in an offline environment, and more recently in an online setting (Edelman et al. 2019). Motor imagery involves the capacity to mentally simulate a movement, which necessitates an internal representation of the movement itself, the environmental limitations, and the sensory outcomes it entails (Munzert et al. 2009). This notion is supported, among others, by research using imaging techniques that show that brain regions involved in action execution are also activated during mental imagery (Hardwick et al. 2018). In fact, the premotor cortex (PMC), supplementary motor areas (Gerardin et al. 2000; Johnson et al. 2002; Kuhtz-Buschbeck et al. 2003; Oostra et al. 2016; Orlandi et al. 2020), parietal regions (Decety et al. 1994; Sirigu et al. 1996; Pelgrims et al. 2009), cerebellum and basal ganglia (Decety et al. 1994; Grealy and Lee 2011; Heremans et al. 2011; Oostra et al. 2016) have been found active in various motor imagery tasks. The PMC and parietal areas would share a functional neural circuitry in the distributed Fronto-Parietal Network (dFPN) (Hétu et al. 2013; Ptak et al. 2017), enabling emulation. This core process specifically handles dynamic motor representations, regardless of the stimulus or output mechanism (Ptak et al. 2017), and does not seem to include the primary motor cortex (Barhoun et al. 2022).

Partial overlap in actual neural activation was observed during the perception of auditory and musical stimuli in musical and auditory imagery. Auditory imagery entails internal, deliberate perception of sounds and music without the need for physical actions or actual auditory stimuli. Studies associate the primarily right-sided activation in frontal and superior temporal regions with auditory imagery (Halpern and Zatorre 1999; Griffiths 2000; Leaver et al. 2009), There is scarce evidence of activation of the primary auditory cortex during musical imagery (Herholz et al. 2012), despite its significant stimulation during musical listening. Furthermore, music-induced emotions would stimulate the dopaminergic nigro-striatal reward-motivation pathway (Blood and Zatorre 2001; Matthews et al. 2020) thus modulating the activity of brain structures associated with emotion and aesthetic appreciation, such as the orbitofrontal cortex (Koelsch 2014) and the nucleus accumbens (Kim et al. 2019).

In contrast, there are not many studies on imagery of social experiences and interactions. Regarding the neural circuits of the ‘social brain’, the literature points to the medial prefrontal cortex, which represents stereotypes, prejudices and social characteristics of people (Proverbio et al. 2017; Ray et al. 2008; Shamay-Tsoory et al. 2009; Tsuchida and Fellows 2012; Proudfit 2015; Molenberghs et al. 2016; Nejati et al. 2021); the insula, which plays a key role in experiencing emotions and processing social cues (Calder et al. 2000; Pugnaghi et al. 2011; Knutson et al. 2013; Boucher et al. 2015; Li et al. 2020); the anterior cingulate cortex (ACC), which is involved in the regulation of emotional and social processes (Hornak et al. 2003; Hadland et al. 2003) and the temporal lobe, which plays an important role in encoding facial expressions, recognising familiar faces and voices, and regulating social behaviour (Toller et al. 2015; Redcay et al. 2016; Ong et al. 2021; Lee Masson and Isik 2021; Reisch et al. 2022; Su et al. 2022).

Regarding the neural correlates of motivational states, the available evidence comes from the field of addiction research. Cravings for substances such as food (Harvey et al. 2005; Asmaro et al. 2012; Wolz et al. 2017; Zorjan et al. 2020; Zapparoli et al. 2022), tobacco (Zinser et al. 1999; McDonough and Warren 2001; Knott et al. 2008; Ferguson and Shiffman 2009; Betts et al. 2021; Tamburin et al. 2021; Gan et al. 2023), alcohol (Herrera-Díaz et al. 2016; Huang et al. 2018), and drugs (Reid et al. 2003; Michel and Koenig 2018; Lin et al. 2022) have been investigated. A recent study revealed the existence of a central craving network, characterised by changes in the activity and functional connectivity of several brain regions, in which limbic regions, together with the pregenual ACC and OBF, may encode the emotional component of associative learning of the paradoxical reward of craving (Huang et al. 2018).

Overall, there is limited knowledge regarding the neural markers of motivational states of non-pathological needs. To examine this matter, a swLORETA investigation was conducted to scrutinize the different patterns of cerebral activations underlying simulated motivational conditions, including “Social Play”, “Music” and “Movement” desires.

These mental states were chosen for several reasons: (i) they were able to clearly and unambiguously modulate the N400 component of ERPs recorded under imagery conditions in a previous experiment by Proverbio and Pischedda (2023a); (ii) they were presumably represented at the cortical level (rather than, for example, in the hypothalamus, which regulates the oemostatic needs of hunger and thirst); and (iii) they reflected realistic rather than fictitious needs that could be easily imagined and experienced by the young adult participants in the EEG experiment. The N400 is an ERP component that is typically evoked in response to conceptually meaningful stimuli (DeLong and Kutas 2020).For example, it is more negative in response to incongruent than congruent words in a sentence, and more negative to unrelated than related words following a prime word. This sensitivity to semantic meanings in relation to an individual’s mental context makes it a reliable index of conceptual representation, particularly interesting for imagery (Gullick et al. 2013) and brain-computer interfaces (Dijkstra et al. 2020).

The EEG data were recorded while participants were requested to vividly imagine distinct motivational states triggered by pictograms. Therefore, individual ERP data underwent independent swLORETA analyses (Palmero-Soler et al. 2007) to determine the intracortical generators of the N400 potentials associated with the three motivational states.

## Materials and methods

### Participants

Twenty right-handed students, (8 males, 12 females), aged 18–35 years (23.20, SE = 1.70) with corrected or normal vision, with no current or history of psychiatric or neurological disorder took part in the study. Inclusion criteria included not having sought treatment for substance misuse, not having any chronic illness and not taking any prescribed medication of any kind. Two participants were excluded from the sample for excessive EEG or ocular artifacts. Participants provided written informed consent. The experiment was conducted in accordance with international ethical standards and Helsinki declaration. The project, entitled “Neurobiological bases of mental reconstruction of visual and auditory stimuli” was pre-approved by the Research Assessment Committee of the Department of Psychology (CRIP) for minimal risk projects, under the aegis of the Ethical committee of University of Milano-Bicocca, on February 3rd, 2020, protocol n: RM-2020-242).

### Stimuli

**Fig. 1:**
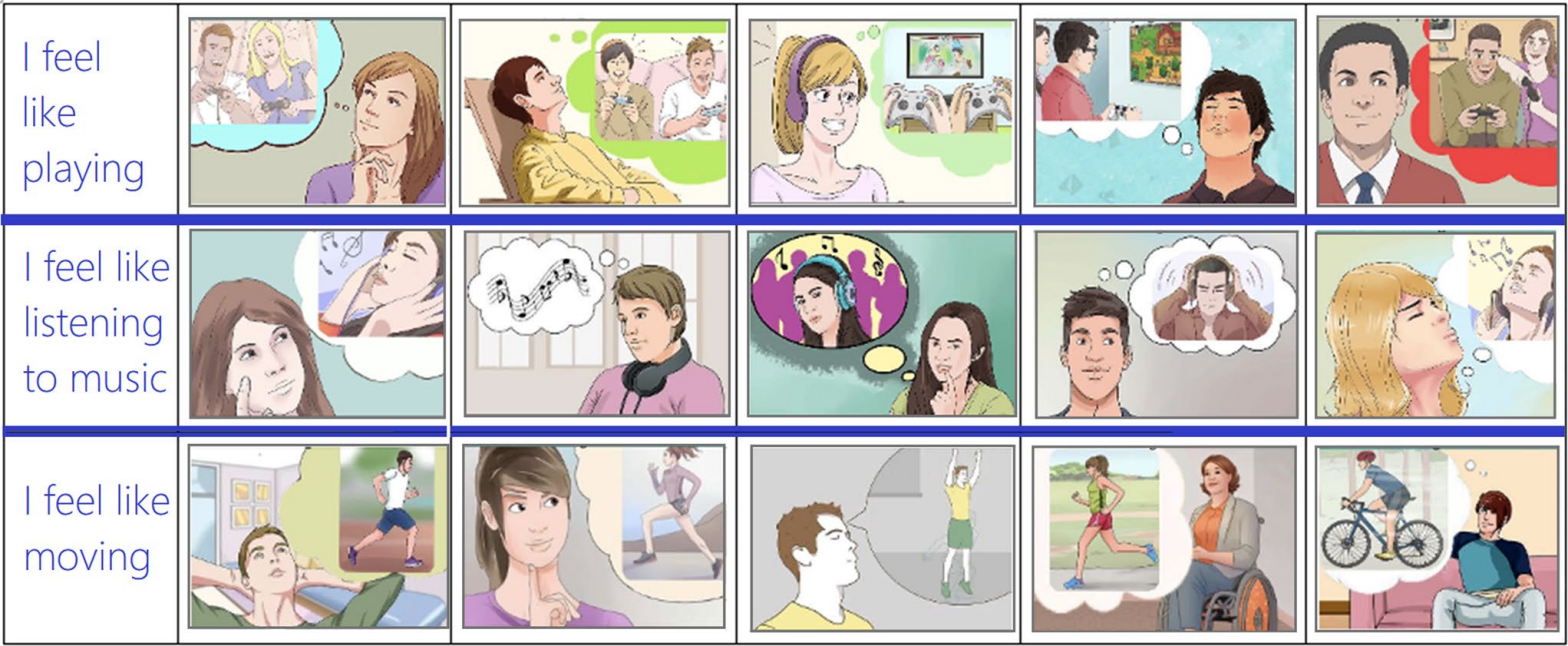
Pictograms used to prompt for the three motivational states were taken from the “P.A.I.N. set” pictionary for assessing individual needs and motivational states in patients who are unable to communicate (Proverbio and Pischedda 2023b)

In the EEG study, pictures taken from a previously validated *Motivational Pictionary* (see Proverbio and Pischedda 2023b for details) were visually shown to the participants to elicit specific motivational states. The stimuli were coloured vignettes (Fig. 1) depicting male and female characters of the apparent age of a university student who, through their facial expressions, facial expressions, context, and use of props, showed clear signs of being in an imagined motivational state of need. Inside a small cloud representing the participant’s thoughts, the fulfilment of the wish was pictorially described. These desires were selected based on questionnaire preliminary administered to a sample of students. The stimuli have been used and tested for their ability to clearly target mental content in a previous electrophysiological study (Proverbio and Pischedda 2023a). The pictograms were correctly classified by 98.4% of the validation participants: the motivational states selected for this study were rated 2.7 (on a scale of 0–3) as easy to imagine, suggesting the reliability of the methodological procedure.

**Fig. 2:**
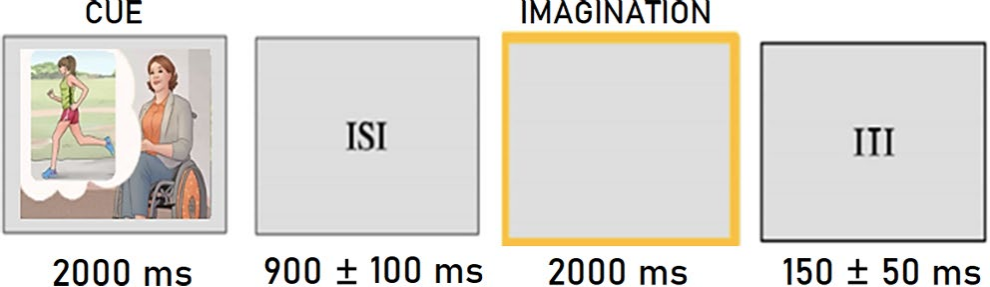
Time sketch of the experimental procedure

Stimuli were presented randomly to each participant in sets consisting of 36 stimuli. Each stimulus lasted for 2000 ms and was followed by an ISI, which consisted of a blank, illuminated screen lasting between 900 ± 100 ms. The ISI was intended to eliminate any after-images on the retina resulting from the prior stimulation (see Fig. 2). A bright yellow frame was presented as a visual prompt for imagery. The frame was located in the corner of the screen against a grey background and lasted 2000 ms. The Inter Trial Interval (ITI) was 150 ± 50 ms. Each stimulus was repeated 6 times in different runs for averaging purposes.

Observers had to maintain focus on a specific point while recording. Participants were given written instructions to recreate the emotional or motivational state associated with the previously viewed image. They were required to keep their gaze fixed on the center of the screen and evoke a subjective feeling based on their own sensations. Prior to the EEG recording, participants attended a concise training session which included two 15-stimulus runs. The session aided the participants in comprehending the task requirements. Importantly, they were not asked to imagine movement, music or play, but to actively evoke a motivational state of craving and desire for the three conditions.

### EEG recording

Brain activity was monitored using 128 electrodes placed according to the 10 − 5 International system, with horizontal and vertical electro-oculograms also being recorded. The averaged mastoids were used as the reference. Electrode impedance was kept below 5 kΩ, and the sampling rate was 512 Hz. EEG and EOG signals were captured using the *Cognitrace* system (ANT Software) and amplified with a bandpass filter (0.16–70 Hz). Any EEG artifacts exceeding ± 50 µV were automatically rejected before averaging. EEG epochs synchronized with stimulus presentation were processed through the *EEProbe* system (ANT Software). ERPs were averaged offline from 100 ms before to 1200 ms after stimulus onset (of prompt for imagery).

The N400 response was quantified in the time window and where it reached its maximum amplitude (in between 400 and 600 ms at anterior frontal sites, for details see Proverbio and Pischedda 2023a). The component was similar to the fronto/polar N400 described in previous literature on imagery-related components (e.g., Schendan and Ganis 2012). It proved to be very sensitive to the motivational state, being larger during imagery related to social play than movement and music.

### Data processing and analyses

To identify the cortical sources of the N400 component in response to simulated motivational scenarios of ‘social play’, ‘music’, and ‘exercise’, three swLORETA models were conducted per participant corresponding to each motivational state. The N400 component was selected in that it proved to be the earliest and most reliable ERP index of category-specific imagery of motivational states, based on Proverbio and Pischedda (2023a) investigation.

Overall, 54 swLORETAs were analysed to identify cortical sources associated with the N400 component elicited by simulated scenarios of “Social Play”, “Music”, and “Movement” motivations. LORETA (Pascual-Marqui et al. 1994, 1999, 2002; Pascual-Marqui 1999, 2002) is an inverse solution that estimates the density of cortical electric current based on measurements taken from the scalp. It utilizes realistic electrode coordinates and is applied to a three-concentric-shell spherical head model, which is registered to a standardized MRI atlas (Talairach and Tournoux 1988). This allows for an approximate anatomical labeling of the neocortical volume. A variant of LORETA, the *standardized Low-Resolution Brain Electromagnetic Tomography* (sLORETA), introduced by Pascual-Marqui (2002), provided additional normalization of results to reduce sensitivity to individual differences in brain anatomy. Later on Palmero-Soler et al. (2007), proposed another method to enhance the sLORETA called swLORETA (the one used in this study), which aimed to address two main challenges: firstly, to overcome the tendency of the linear inverse procedure to reconstruct sources near the sensor location and secondly, to reduce the solution’s sensitivity to noise in the data. This novel approach had shown superior performance compared to sLORETA, particularly in noisy conditions and for reconstructing deep sources (Palmero-Soler et al. 2007). SwLORETA offers improved accuracy and robustness in inverse solutions (e.g. Boughariou et al. 2015).

SwLORETA is a brain imaging technique that provides subset of 1056 electromagnetic dipoles, providing information on their magnitude of activation (in nA) and tridimensional coordinates in the cerebral space (Talairach and Tournoux 1988). In order to statistically analyze the large amount of data, and differentiate the specific sources of brain activation across the various motivational states, 8 regions of interest (ROIs) per hemisphere were identified following the ROI clustering procedure used to perform statistical analyses on individual LORETA solutions (Babiloni et al. 2004, 2006; Cannon et al. 2008, 2009). The selected ROIs are listed in Table 1.

**Table 1:** List of regions of interest identified and referenced to the Gyri and Brodmann areas (BAs) included in each cluster

ROIs	BA	GYRUS
*OCC*	18, 19	Inferior Occipital Gyrus
(Occipital Cortex)		Middle Occipital Gyrus
		Superior Occipital Gyrus
		Lingual Gyrus (also BA 17)
		Cuneus (also BA 17)
*FUSIF*	19, 20, 37	Fusiform Gyrus
(Fusiform Area)		Cerebellum, Anterior Lobe
		Posterior lobe, Declive
		Middle occipital gyrus (only BA 37)
		Inferior temporal gyrus (only BA 37)
*TEMP*	19, 20, 21, 22, 38, 39, 41, 42	Superior Temporal Gyrus
(Superior, Middle and Inferior Temporal Cortex)		Middle Temporal Gyrus
		Inferior Temporal Gyrus
*PREM*	4, 6	Precentral Gyrus (also BA 43)
(Premotor Cortex)		Middle Frontal Gyrus
		Medial Frontal Gyrus
		Superior Frontal Gyrus
		Precentral Gyrus
		Paracentral Lobule
*FRONTAL*	8, 9, 46	Middle Frontal Gyrus
(Medial, Superior Frontal and Dorsolateral Prefrontal Cortex)		Medial Frontal Gyrus
		Superior Frontal Gyrus
*ORB\IF*	10, 11, 44, 45, 47	Superior Frontal Gyrus
(Orbitofrontal and Inferior Frontal Cortex)		Medial Frontal Gyrus
		Middle Frontal Gyrus
		Inferior Frontal Gyrus
		Rectal Gyrus
*PARIETAL*	1, 2, 3, 7, 19, 39, 40	Superior Parietal Lobule
(Parietal Cortex)		Inferior Parietal Lobule
		Precuneus
		Postcentral Gyrus
		Supramarginal Gyrus
		Angular Gyrus
*LIMBIC*	20, 23, 24, 28, 31, 34, 35, 36, 38	Uncus
(Limbic Area)		Cingulate Gyrus
		Anterior Cingulate
		PPA

**Fig. 3:**
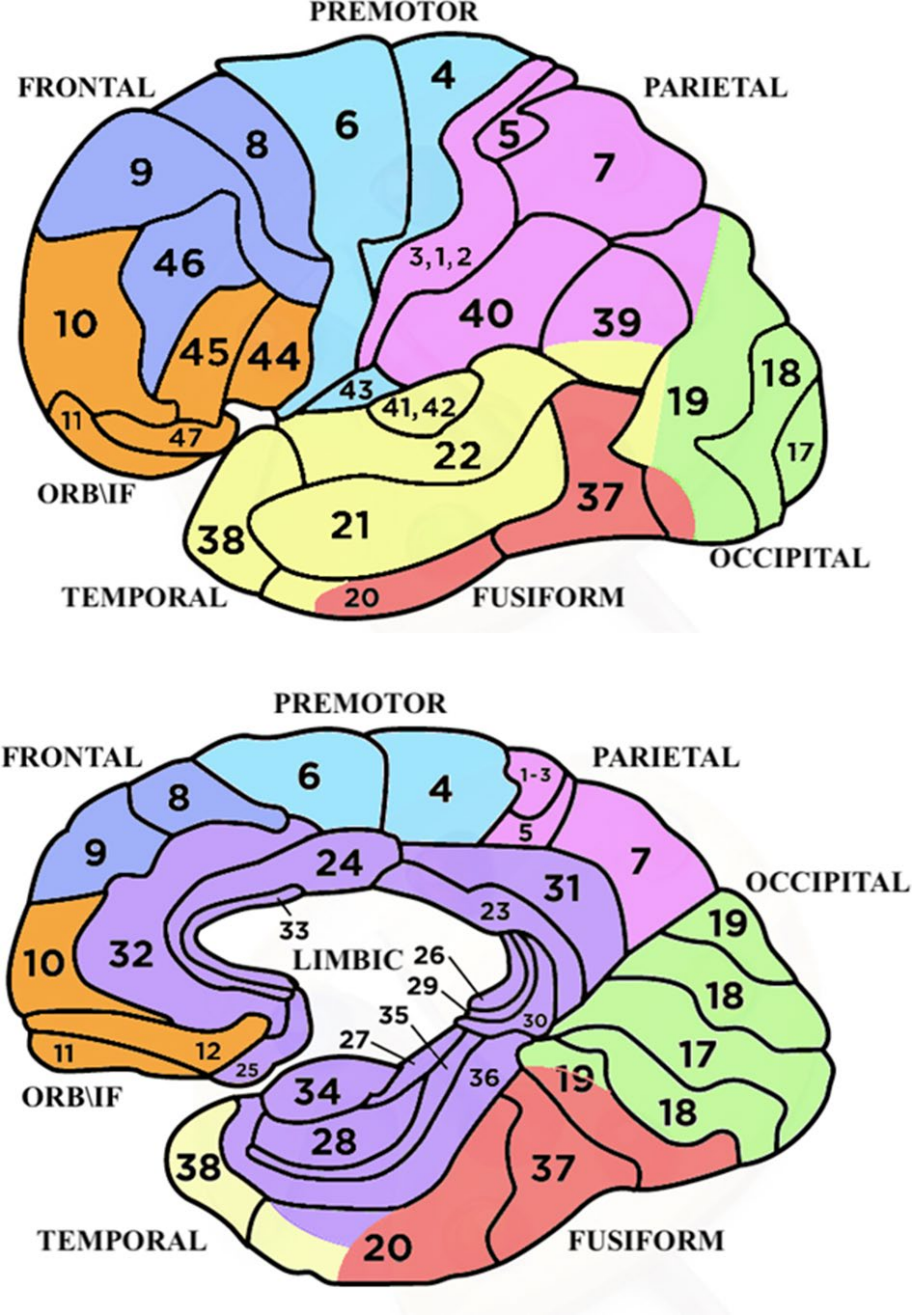
Anatomical subdivision of chosen regions of interest (ROIs) are displayed in an external side view (top) and sagittal view (bottom). Occipital areas are in green, fusiform in red, temporal in yellow, premotor in light blue, frontal in blue, OBF/IF in orange, parietal in pink, limbic in purple

The designed ROIs, illustrated in Fig. 3, were the following ones: Occipital areas (OCC), Fusiform gyri (FUSIF), Superior, Middle and Inferior Temporal areas (TEMP), Parietal areas (PARIETAL), Limbic areas (LIMBIC), Premotor areas (PREM), Medial, Superior Frontal, and Dorsolateral Prefrontal areas (FRONTAL) and Orbitofrontal and Inferior-Frontal areas (OBF/IF). After initial analysis of all ROIs, the data relative to PARIETAL and LIMBIC ROIs were not included in the statistical analyses due their poorly differentiated values across the different motivational states examined. Indeed, they are known to be always active during imagery of emotional relevant content, regardless of the specific motivational state (Antons et al. 2023; Sambuco et al. 2020).

As each ROI sometimes had more than one active dipole, the dipole with the highest magnitude in a given hemisphere was identified, and then the dipole with the highest magnitude in the corresponding homologous brain area was identified. Priority was given to homologous areas (same BA and same gyrus), but if this was not possible, the dipole with the highest magnitude in the opposite hemisphere within the ROI considered was selected. For statistical analysis, those homologous areas that were not active according to swLORETA were assigned a value of 0.50 nA (i.e., below statistical threshold).

A three-way repeated measures ANOVA was applied to the individual magnitudes of activation of the different active sources (in nA) recorded in the N400 latency range in the different imaginary states. Factors were: MOTIVATIONAL STATE (3 levels: social play, music, movement), ROI (6 levels: OCC, FUSIF, TEMP, PREM, FRONTAL, OBF/IF) and HEMISPHERE (2 levels: left, right). Fisher and Tukey post-hoc comparisons were used to assess differences between means.

In addition, the Wilcoxon signed-rank test was used to compare the strength of the N400 electromagnetic sources in the brain areas of interest. More detailed analyses were performed for activations recorded in the temporal, OBF/IF and pre-motor ROIs, in both left and right hemispheres, across the motivational states of ‘social play’, ‘music’ and ‘movement’.

## Results

Individual swLORETAs were applied to individual N400 voltages (400–600 ms) recorded during the three motivational states. A list of all active electromagnetic dipoles, recorded for each individual as a function of the ROI and the motivational state considered is reported in Supplementary file 1. The resulting individual neuroimages are shown in the results section. The statistical analyses applied to the individual dipole strengths (in nA) recorded as a function of motivational state, ROI and cerebral hemisphere are reported below.

### Hemisphere

**Fig. 4:**
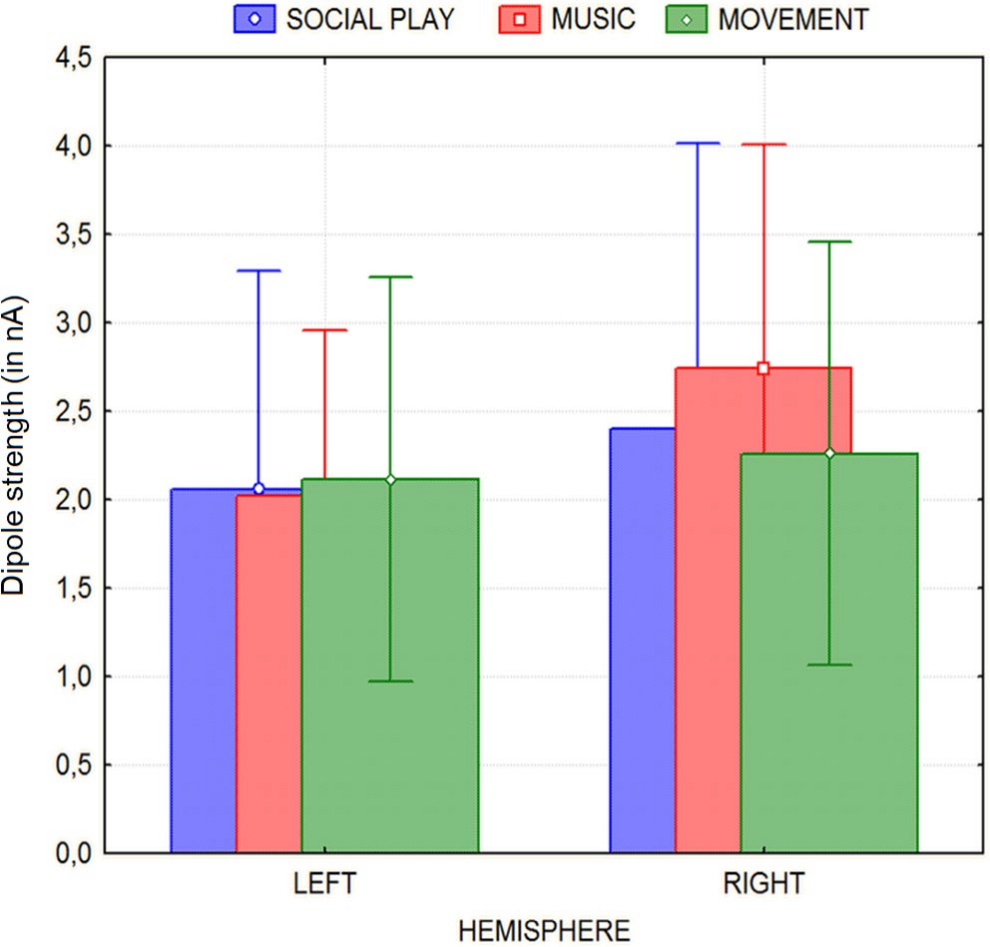
Strength of electromagnetic signals (along with SD values) recorded over the left and the right hemispheres during the three motivational states

The ANOVA revealed the significant effect of hemisphere [F (1,17) = 6.06, p < 0.02], with greater magnitudes recorded over the right hemisphere (M = 2.47 nA, SE = 0.23) than over the left hemisphere (M = 2.06 nA, SE = 0.17), as shown in Fig. 4.

### ROI

The ROI factor was also significant [F (5.85) = 5.05, p < 0.001], with greater amplitude over the temporal area (M = 2.78 nA, SE = 0.30), independent of motivational state. Post-hoc comparisons showed no significant difference in the activations recorded in the fusiform (M = 2.56 nA, SE = 0.32), occipital (M = 2.52 nA, SE = 0.26) and premotor (M = 2.11 nA, SE = 0.18) ROIs. Intermediate brain activation was observed in the OBF/IF area (M = 1.94 nA, SE = 0.26) (p < 0.03), while the smallest activity was recorded in the frontal area (M = 1.66 nA, SE = 0.17), which differed from the temporal (p < 0.001), fusiform (p < 0.01) and occipital (p < 0.02) activations.

### Motivational state

The main factor Motivational State was not significant per se [F (2,34) = 0.76, p = 0.48], suggesting an anatomical specificity of brain activations depending on the three motivational states.

### Motivational state x ROI

**Fig. 5:**
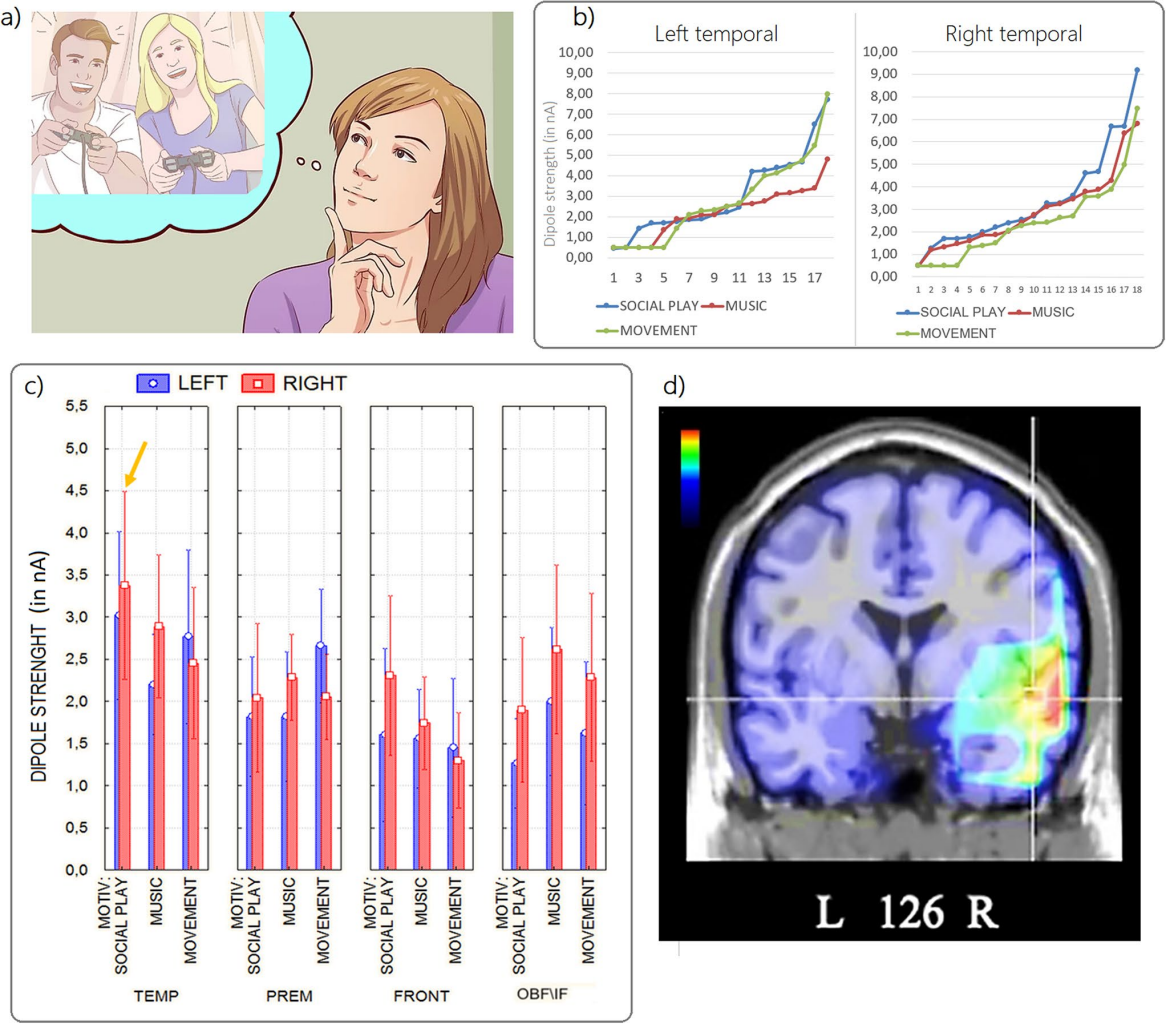
**a**) Example of pictogram used to prompt the social play desire; **b**) individual data relative to dipole strengths recorded within the left and right temporal ROIs as a function of motivational state; **c**) mean values of N400 power recorded as a function of the ROI, cerebral hemisphere and motivational state; **d**) coronal view of swLORETA source reconstruction of N400 surface potentials recorded in the 400–600 ms time window during the social play motivational state (group data). Group LORETAs were performed on grand-average ERPs. The various colours represent differences in the magnitude of the electromagnetic signal (nA), with brighter colours (from orange to red) indicating maximum strength, and the darkest colours (from blue to black) indicating a value of 0. The electromagnetic dipoles appear as arrows and indicate the position, orientation and magnitude of the dipole modelling solution applied to the ERP waveform in the specific time window. L, left; R, right; numbers refer to the displayed brain slice in the coronal MRI imaging plane: from 1 to 217, where 18 is the most posterior cortical slice and 217 is the most anterior. Cortical slice numbering excluded MRI slices not containing cortex

**Fig. 6:**
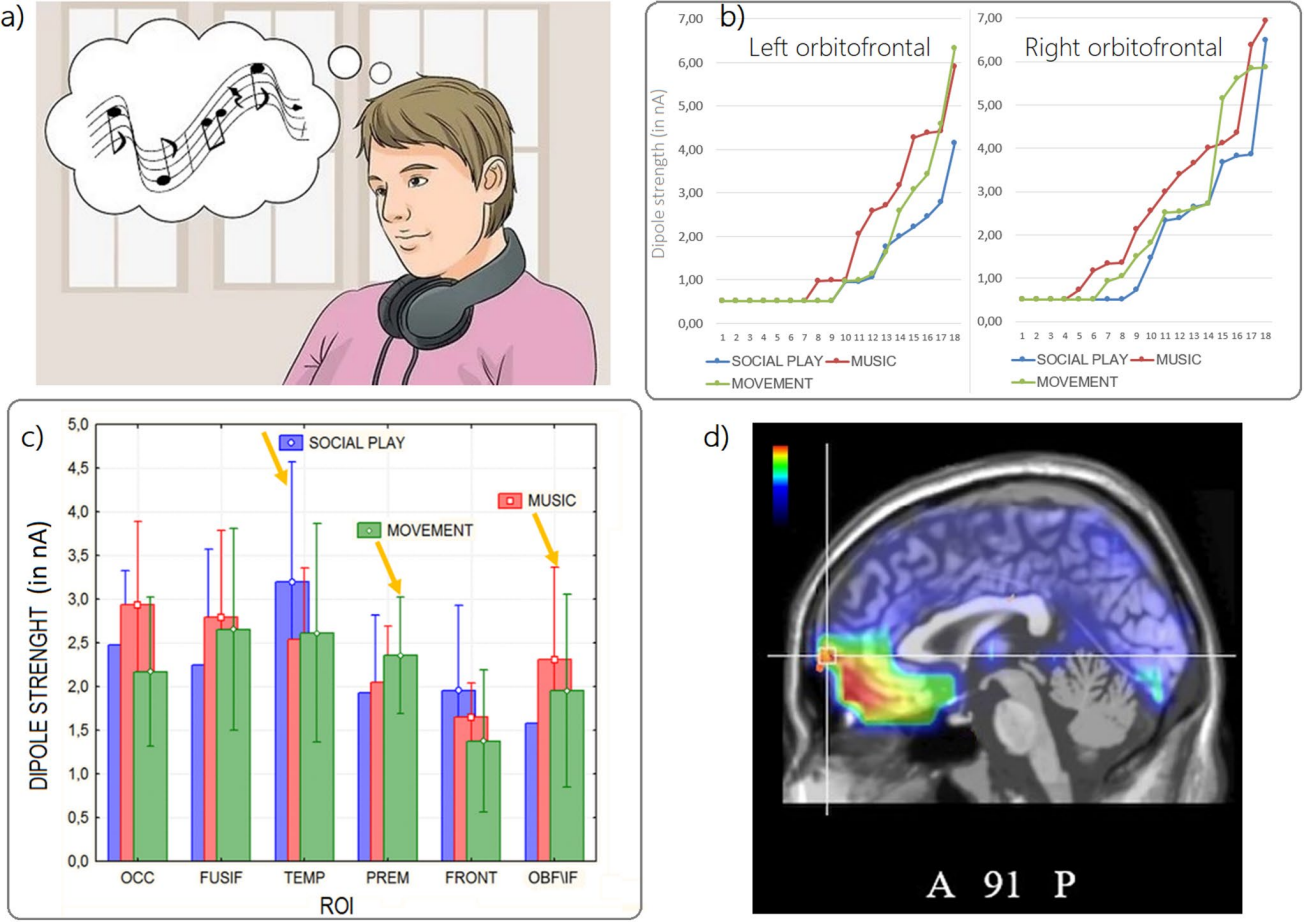
**a**) Example of pictogram used to prompt the music listening desire; **b**) individual data relative to dipole strengths recorded within the left and right OBF/IF ROIs as a function of motivational state; **c**) mean values of N400 power recorded as a function of the ROI and motivational state but averaged across hemispheres; **d**) sagittal view of swLORETA source reconstruction of N400 surface potentials recorded in the 400–600 ms time window during the music listening motivational state (group data). Group LORETAs were performed on grand-average ERPs. The various colours represent differences in the magnitude of the electromagnetic signal (nA), with brighter colours (from orange to red) indicating maximum strength, and the darkest colours (from blue to black) indicating a value of 0. The electromagnetic dipoles appear as arrows and indicate the position, orientation and magnitude of the dipole modelling solution applied to the ERP waveform in the specific time window. P, posterior; A, anterior; numbers refer to the displayed brain slice in the sagittal MRI imaging plane: from 1 to 181, where 1 is the rightmost cortical slice and 181 is the leftmost slice

Indeed, the ANOVA showed the significant interaction of motivational state x ROI [F (10,170) = 2.30, p = 0.01]. Tukey post-hoc comparisons showed that the temporal area was the most active ROI during the Social Play motivational state (M = 3.20 nA, SE = 0.46) compared to all other areas: Premotor (M = 1.93 nA, SE = 0.30, p = 0.006), Frontal (M = 1.95 nA, SE = 0.33, p = 0.009), OBF/IF (M = 1.58 nA, SE = 0.18, p < 0.001), Occipital (M = 2.48 nA, SE = 0.28, p = 0.022) and Fusiform (M = 2.25 nA, SE = 0.44, p = 0.003) areas. Furthermore, the Temporal ROI was more active during the Social Play state than the Music Listening state (M = 2.54 nA, SE = 0.27, p = 0.04), and as a trend (p = 0.06) also with respect to the Movement motivational state (M = 2.61 nA, SE = 0.42). This difference reached full significance over the right temporal cortex (p = 0.03), as shown by simple effects (“Social Play” M = 3.37 nA, SE = 0.53 vs. “Movement” M = 2.45 nA, SE = 0.42) (see Fig. 5). Posterior brain areas were more active during the Music motivational state, particularly the occipital (M = 2.94 nA, SE = 0.32; p < .001), fusiform (M = 2.79 nA, SE = 0.33; p < .001), and temporal (M = 2.54 nA, SE = 0.27; p = .005) ROIs. Simple effect analyses (within the two hemispheres) showed that the hemispheric asymmetry in brain activation was particularly pronounced during the Music motivational state, with stronger right hemisphere activation (M = 2.74 nA, SE = 0.24) compared to the left (M = 2.02 nA, SE = 0.18). The more prominent ROI for music listening was the bilateral OBF/IF (M = 2.30 nA, SE = 0.36), as shown in Fig. 6. Post-hoc comparisons showed that the OBF/IF area was bilaterally more active (p = 0.02) during ‘music’ (M = 2.31 nA, SE = 0.36) than ‘social play’ (M = 1.58 nA, SE = 0.18) and ‘movement’ (M = 1.95 nA, SE = 0.37), motivational states.

**Fig. 7:**
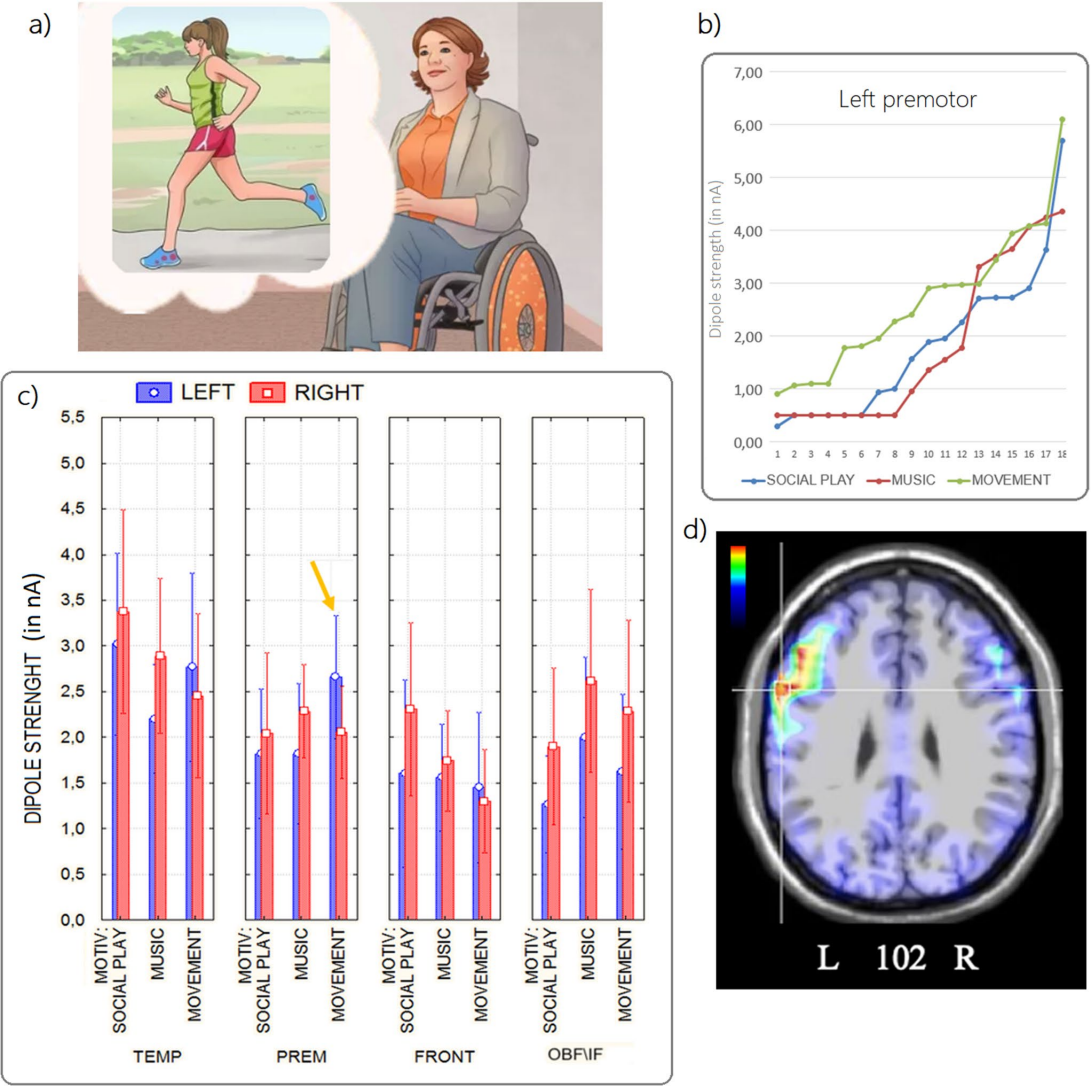
**a**) Example of pictogram used to prompt the movement desire; **b**) individual data relative to dipole strengths recorded within the left premotor ROI as a function of motivational state; **c**) mean values of N400 power recorded as a function of the ROI, cerebral hemisphere and motivational state (the data are also shown in Fig. 5c); **d**) axial view of swLORETA source reconstruction of N400 surface potentials recorded in the 400–600 ms time window during the movement motivational state (group data). Group LORETAs were performed on grand-average ERPs. The various colours represent differences in the magnitude of the electromagnetic signal (nA), with brighter colours (from orange to red) indicating maximum strength, and the darkest colours (from blue to black) indicating a value of 0. The electromagnetic dipoles appear as arrows and indicate the position, orientation and magnitude of the dipole modelling solution applied to the ERP waveform in the specific time window. L, left; R, right; numbers refer to the displayed brain slice in the axial MRI imaging plane: from 1 to 181, where 1 is the deeper cortical slice (inferior) and 163 is the shallower (superior). Cortical slice numbering excluded MRI slices not containing cortex

Finally, the pre-motor ROI was more active during movement (M = 2.36 nA, SE = 0.22) than during the other motivational states (music: M = 2.05 nA, SE = 0.21; social play: M = 1.93 nA, SE = 0.30), as shown in Fig. 7. The pre-motor cortex (M = 2.36 nA, SE = 0.23) was significantly more active (p < 0.001) than the frontal ROI (M = 1.37 nA, SE = 0.27) during the motivational state “Movement”. Activation in the pre-motor cortex (M = 2.36 nA, SE = 0.23) was significantly greater (p < 0.001) than activation in the frontal cortex (M = 1.37 nA, SE = 0.27) during the movement motivation state. The frontal area was found to be the least active during this motivational state (p < 0.01), with a significantly lower magnitude (nA) compared to the following areas Occipital (M = 2.17 nA, SE = 0.29), Fusiform (M = 2.65 nA, SE = 0.39), Temporal (M = 2.61 nA, SE = 0.42). Overall, a simple effects analysis revealed a hemispheric asymmetry in favour of the left hemisphere, with a stronger activation (p = 0.05) of the left premotor area during “Movement” (M = 2.66 nA, SE = 0.32) than during “Music” (M = 1.82 nA, SE = 0.36) and “Social Play” motivational states (M = 1.82 nA, SE = 0.34).

### Non-parametric tests

Wilcoxon signed-rank test was performed to more closely compare specific subsets of activations in specific brain areas, across motivational states, by focusing on ROIs that were found by ANOVA to be distinctive for a motivational state. The difference in strength of N400 electromagnetic sources (in nA) between the motivational states “Social Play”, “Music” and “Movement” with a focus on the Temporal, OBF/IF and Premotor ROIs in the two cerebral hemispheres, respectively, was assessed through this test.

### Temporal ROI

Individual activations recorded in the left Temporal ROI were stronger in the “Social Play” condition than in the “Music” condition [Wilcoxon signed-rank test: Z (17) = 2.34, p < 0. 02], whereas there was no difference with the Movement condition [Wilcoxon signed-rank test: Z (17) = 1.32, p = 0.19]. However, individual activations recorded in the right Temporal ROI were significantly greater in the “Social Play” condition than in the “Music” condition [Wilcoxon signed-rank test: Z (17) = 3.52; p < 0.001] and “Movement” [Wilcoxon signed-rank test: Z (17) = 3.62, p < 0.001] motivational states. This area provides to be a reliable marker of the social play motivational states across participants, with some variability (see Fig. 5b).

### OBF/IF ROI

For the left OBF/IF area the non-parametric tests revealed significant differences in N400 dipole strength between “Music” and “Social Play” conditions [Wilcoxon signed-rank test: Z (11) = 2.93, p = 0.003] and also between “Music” and “Movement” motivational states (as can be appreciated in Fig. 6b) [Wilcoxon signed-rank test: Z (11) = 2.49, p = 0.013].

For the right OBF/IF area the non-parametric tests revealed significant differences in N400 dipole strength between “Music” and “Social Play” conditions [Wilcoxon signed-rank test: Z (14) = 3.29, p < 0.001] and as a strong trend between “Music” and “Movement” motivational states [Wilcoxon signed-rank test: Z (14) = 1.85, p = 0.06].

### Premotor ROI

For the left Premotor area the non-parametric tests revealed significant differences in N400 dipole strength between “Movement” and both “Social Play” [Wilcoxon signed-rank test: Z (18) = 3.72, p < 0.001] and “Music” [Wilcoxon signed-rank test: Z (18) = 3.29, p = 0.001] motivational states. This can be appreciated in Fig. 7b. For the right Premotor area the non-parametric tests did not reveal any statistically significant differences in N400 dipole strength between “Movement” and both “Social Play” [Wilcoxon signed-rank test: Z (15) = 0.62, p = 0.53] and “Music” [Wilcoxon signed-rank test: Z (16) = 1.53, p = 0.13] motivational states.

**Fig. 8:**
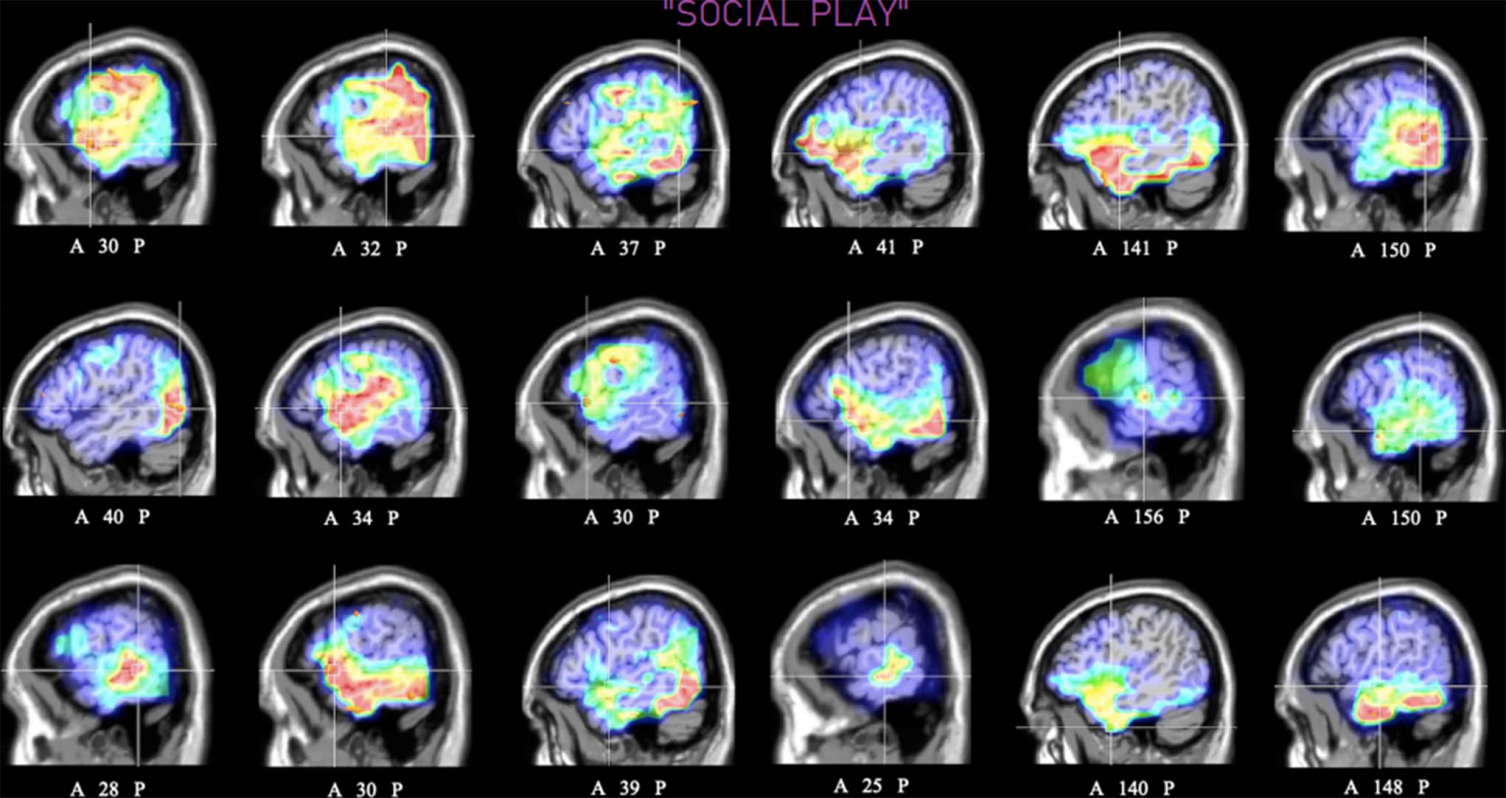
Sagittal view of individual swLORETA source reconstructions of electromagnetic signals recorded during the social play motivational state (N = 18). Numbers refer to the displayed brain slice in the sagittal MRI imaging plane: from 1 to 181, where 1 is the rightmost cortical slice and 181 is the leftmost slice. Twelve out of eighteen participants displayed a right hemispheric asymmetry in the activation of the temporal ROI. Overall, 100% of participants displayed a temporal activation in this motivational state

**Fig. 9:**
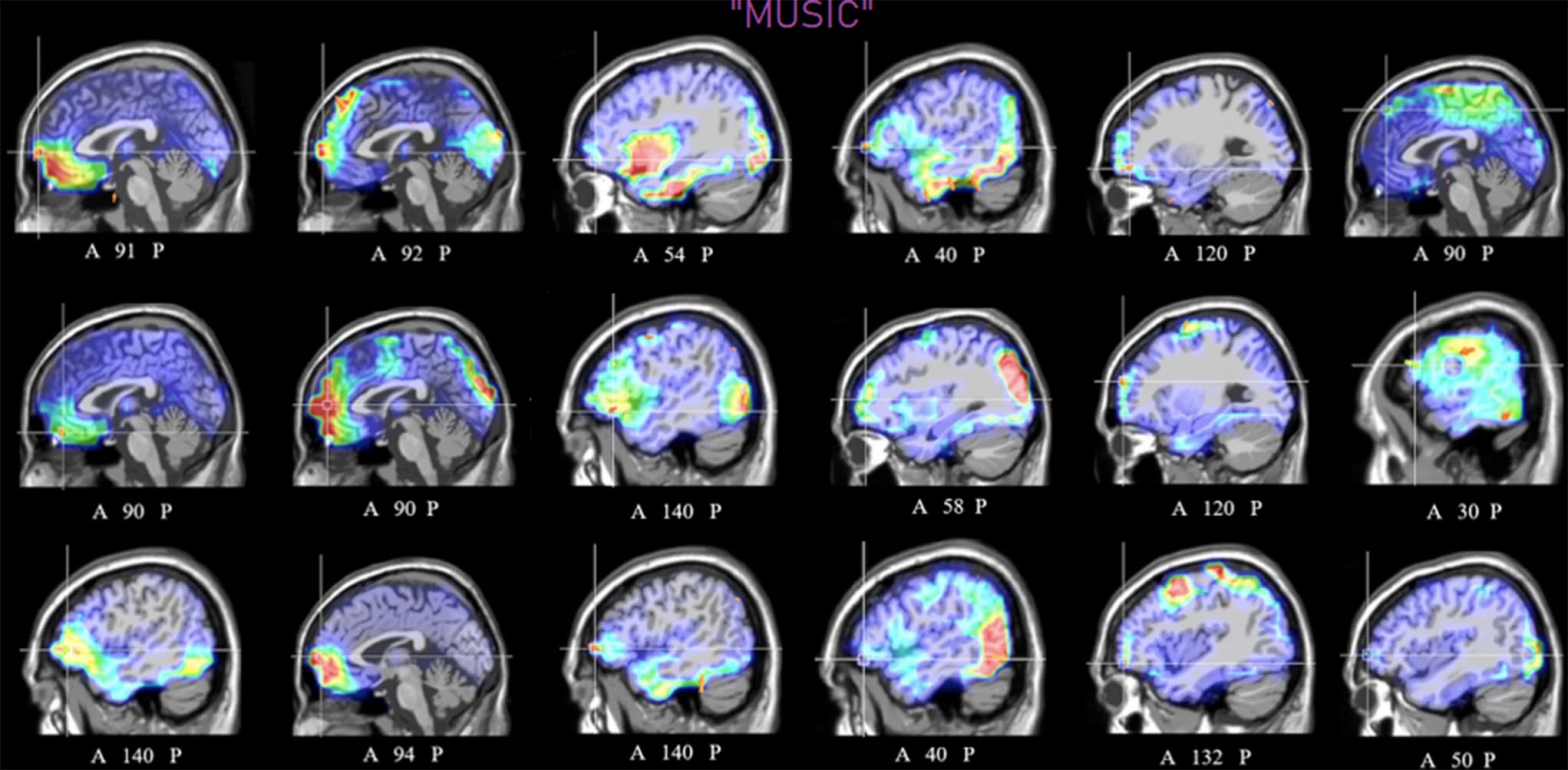
Sagittal view of individual swLORETA source reconstructions of electromagnetic signals recorded during the music listening motivational state (N = 18). Numbers refer to the displayed brain slice in the sagittal MRI imaging plane: from 1 to 181, where 1 is the rightmost cortical slice and 181 is the leftmost slice. Overall, 100% of participants displayed an OBF/IF activation in this motivational state

**Fig. 10:**
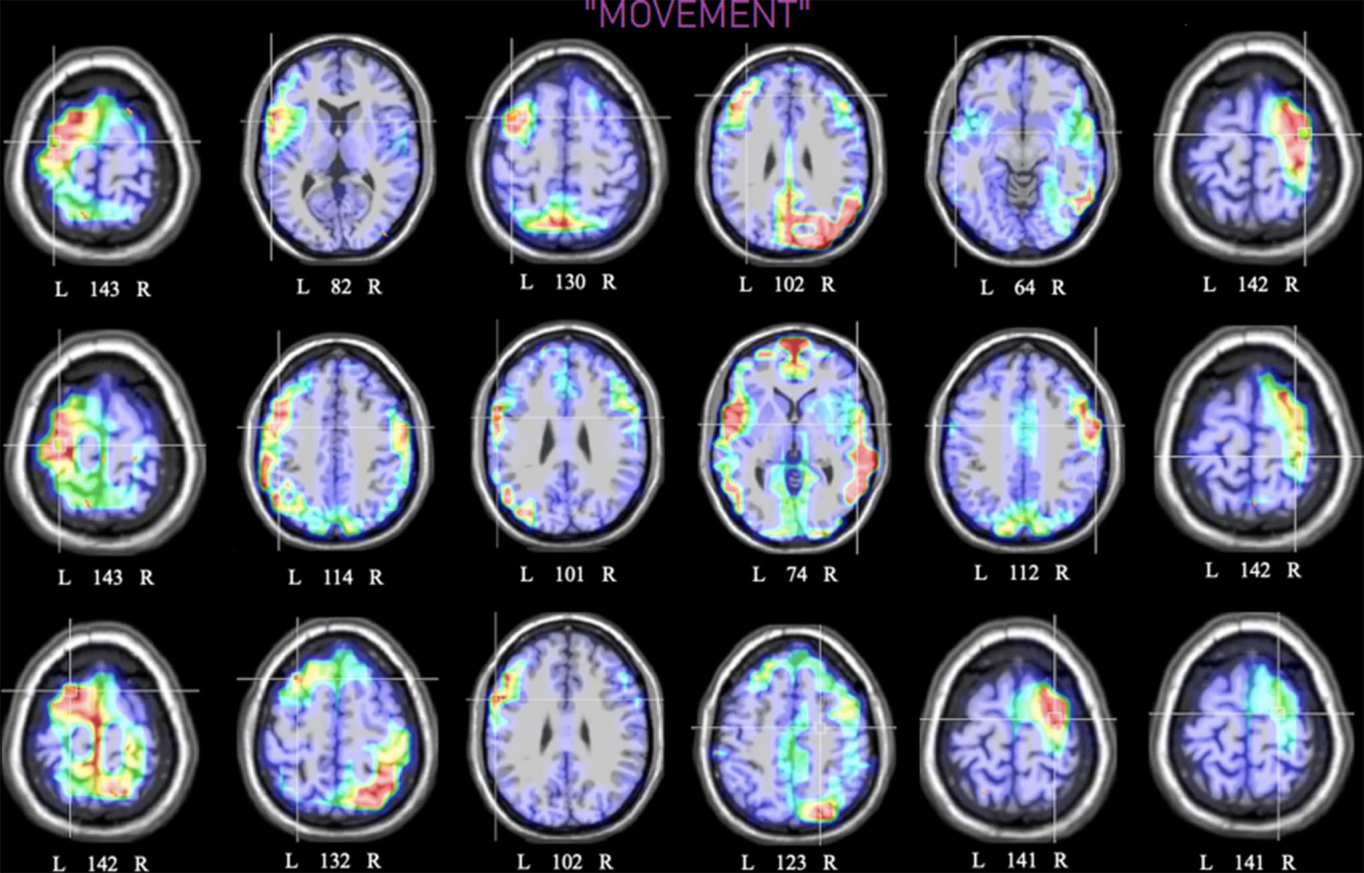
Axial view of individual swLORETA source reconstructions of electromagnetic signals recorded during the movement motivational state (N = 18). Numbers refer to the displayed brain slice in the axial MRI imaging plane: from 1 to 181, where 1 is the deeper cortical slice (inferior) and 163 is the shallower (superior). Eleven out of eighteen participants displayed a left hemispheric asymmetry in the activation of the premotor ROI. Overall, 100% of participants displayed a premotor activation in this motivational state

Despite interindividual variability, swLORETA Tomography Solutions supported statistical analyses of dipole strength for the N400 component of ERP recorded during different motivational states. The present investigation focuses on the Temporal ROI activation, prevalent during the “Social Play” motivational state (Fig. 8), the OBF/IF ROI activation prevalent during the “Music” state (Fig. 9), and the Premotor ROI activation prevalent during the “Movement” state (Fig. 10).

During the imaginary desire state to “Socially Play” with friends, the swLORETA tomographic analyses of the individuals’ brain function (see Fig. 8) mostly revealed a bilateral neural activity in the temporal area. However, most participants (12 out of 18) displayed a right hemispheric asymmetry in the temporal activation. While consistently exhibiting substantial individual variances, activations can be also observed in the frontal-premotor and parietal areas.

During the imagined desire to listen to music, the swLORETA tomographic solutions of each subject (see Fig. 9) demonstrated a focus of activity in the OBF/IF region of interest, while still displaying significant individual differences in the extent of activation. Additionally, common activation was found in the temporal, occipital, and frontal regions, with higher activity observed in the right hemisphere. Parietal activation was also detected in some subjects.

During the recalled desire for movement, the swLORETA tomographic solutions of individual subjects (see Fig. 10) demonstrated strong bilateral activation of premotor and temporal areas, alongside less pronounced activations in the occipital and parietal regions. Individual differences persisted. However, most participants (11 out of 18) showed activation in the left premotor ROI, while it was on the right side for the remaining participants.

## Discussion

The aim of the present study was to investigate brain signals associated with imaginary motivational states, thus possibly identify reliable neurocognitive markers of distinct mental states. Individual source reconstructions of neural activity, based on the N400 component of ERPs, were performed in relation to three frequently occurring secondary needs of young adults: “Social Play”, “Music” and “Movement”. Contrasting sources of reconstruction on an individual data level is notably novel, although this approach has been previously implemented in BCI research (e.g. Babiloni et al. 2004, 2006; Cannon et al. 2008, 2009).

Both Kosslyn et al. (2001) and McNorgan (2012) (referring to mental imagery), and Kavanagh et al. (2005) (referring to motivational states) postulated that imaginary states would activate distinct brain regions, somewhat akin to those activated during corresponding sensory or functional processing, in a category-specific way. Indeed, while Kosslyn et al. (2001) and McNorgan (2012) argue that the neural processes responsible for perceiving sensory information within a specific modality also come into play during the process of mental imagery, Kavanagh et al. (2005) highlight the decisive role of imagery in desire sensation, as it replicates sensory and emotional aspects of target experiences.

The main effect analysis of this study demonstrated a right hemispheric asymmetry in brain activation, irrespective of motivational states and ROIs. However, the asymmetry was more pronounced for music imagery with a greater magnitude of N400 sources in the right hemisphere than the left. Consistently, previous studies have reported a right hemispheric asymmetry for tone imagery (Guo and Chen 2022), visuomotor imagery (Kwon et al. 2023), spatial navigation imagery (Boly et al. 2007), facial expression imagery (Kim et al. 2007), emotional imagery (Tomasino et al. 2014) and music imagery (Zatorre and Samson 1991; Zatorre and Halpern 1993; Halpern 2001). On the flip side, a tendency towards left hemispheric asymmetry would be usual for imagining movement (Zou et al. 2022), imagining written language (see the review by Liu et al. 2022) or tools (Belardinelli et al. 2009).

In addition to the activation of distinct ROIs for each motivational state, a set of common areas were found to be involved during the imaginary motivational states and could be identified as their neural substrates. These areas mostly included visual, parietal and prefrontal cortices, which were particularly active during the imaginary states, as predicted based on previous literature (McNorgan 2012; Winlove et al. 2018; Dijkstra et al. 2019; Chen et al. 2021). Indeed, the parietal and prefrontal regions have been recognized for their part in short-term memory, allowing for the retention and manipulation of information (Chen et al. 2021); an essential component for mental imagery. Most importantly, midline cortical structures belonging to the *default mode network* (Horn et al. 2013; Buckner et al. 2008) such as medial prefrontal cortex (Frontal ROI), the posterior cingulate cortex (Limbic ROI), the precuneus and angular gyrus (Parietal ROI) were found often active during all motivational states, as shown by a preliminary analysis with all ROIs (see also the Supplementary file), which is congruent with their role in self-referential processing, self-reflection, day-dreaming, and especially emotion of one’s self, i.e., reflecting about one’s own emotional state, which corresponded precisely to task requirements.

### Social play

Overall, the Temporal ROI was more active during the “Social Play” than the other two imagery states, especially over the right hemisphere. It is noteworthy that the Temporal region (including the Superior Temporal, Middle Temporal, and Inferior Temporal Gyri), was the most active brain area, in the “Social Play” condition, with respect to the other ROIs.

The literature has emphasized the role of this area in processing social experiences (Haruno and Kawato 2009; Shinoura et al. 2011; Toller et al. 2015; Ong et al. 2021; Su et al. 2022), with the temporal region playing a key role in social memory and the retrieval of information pertaining to prior social experiences (Okuyama et al. 2016), Theory of Mind or mentalization during human interactions (Frith and Frith 2003), facial expression interpretation (Reisch et al. 2022), and visualization of friends’ faces and voices (Lee Masson and Isik 2021. The concept of social play is not exclusive to humans; as illustrated in the review by Kellman and Radwan (2022), it is an innate and universal behaviour that represents an intrinsic need for all mammals, allowing individuals to cultivate their social, cognitive and communicative skills. Indeed, Ong et al. (2021) have shown that a particular group of neurons in the STS signal the rewards procured by social and cooperative behaviour in monkeys, while Haruno and Kawato (2009) demonstrated how the STS plays a significant role in social and interactive play contexts. The participation of this area could also stem from its function in giving prognostic clues about the conduct of others (Frith 2007). In summary, the main activation of the Temporal area during social play desire corresponds to prior literature that links this region with various aspects of social interactions, specifically within the right hemisphere.

### Music

During listening to “Music” motivational state a pronounced right-sided asymmetry in brain activation was found regardless of the ROI considered, which is consistent with earlier research indicating a right lateralization for music processing and music imagery (Halpern and Zatorre 1999; Halpern et al. 2004; Herholz et al. 2012). Among other evidences, there is a documented case where a patient with a right hemisphere infarction, affecting the frontal and anterior temporal areas, experienced musical hallucinations, which clearly points to a role of the right hemisphere in the control and development of musical imagery (Buchwald et al. 2020).

As the temporal ROI was also highly engaged in the “social play” state, the OBF/IF region was found to be the most distinctive ROI characterising the “Music” motivational state, exhibiting greater activity during “Music” than the other two states. In the auditory imagery field, several studies have shown how the IFG and the PFC (Zatorre et al. 1996; Yoo et al. 2001; Leaver et al. 2009; Herholz et al. 2012; Lima et al. 2015) along with the PMC and the secondary auditory cortex are strongly involved in music imagery. Interestingly, Griffiths (2000) found a correlation between musical hallucinosis and the inferior frontal cortices, temporal lobes, basal ganglia, and the cerebellum. In examining the more emotional aspect of music, other investigations have shown how the optimal groove, sense of rhythm, flow, and pulsation that is perceived in a musical piece, are able to activate OBF and the NAc, regions that are important components of the reward network (Matthews et al. 2020). Research has shown that music-induced emotions activate the reward-motivation circuit (Blood and Zatorre 2001) and can modulate the activity in brain structures linked to emotions, including the OBF (Koelsch 2014). Moreover, Huang et al. (2018) proposed that the OBF, along with the limbic regions and the pregenual ACC, play a central role in the emotional component of a central craving network. It is evident how the longing and expectation for the enjoyment of one’s preferred music could activate the OBF cortex distinctively for music desire. Moreover, during music motivational state, it was possible to observe the activation of the temporal area especially over the right hemisphere. This finding is in line with the previous literature the neural basis of both music perception (Warren et al. 2003; Warren 2008) and imagery (Zatorre et al. 1996; Halpern 2001; Yoo et al. 2001; Halpern et al. 2004; Herholz et al. 2012; Buchwald et al. 2020).

### Movement

One of the more active areas, and the most distinctive, for the “movement” motivational state was the premotor cortex, with a general left hemispheric asymmetry. Indeed the left premotor cortex (PMC) was more active during desire for “movement” than the other two imagery states. Several studies have highlighted the crucial role of the premotor region during movement execution, specifically in the controlling and learning goal-oriented actions (Mochizuki et al. 2005; Beurze et al. 2007; Cross et al. 2007; Rizzolatti and Sinigaglia 2010), especially in the left hemisphere (Schluter et al. 2001; Johansen-Berg et al. 2002; Rushworth et al. 2003). In the motor imagery field, the functional role of the PMC is supported by evidence indicating that stroke patients with an intact PMC retain their motor imagery capabilities (Johnson et al. 2002). Furthermore, several studies have documented the involvement of the PMC region during motor imagery tasks (Decety et al. 1990, 2004; Guillot et al. 2009; Lorey et al. 2009; Gao et al. 2011; Oostra et al. 2016).

In the present study, participants were instructed to imagine the urge to move (which was made easier by the circumstance that they had to maintain absolute immobility for EEG recording purposes). Therefore, it can be assumed that they impersonated this desire from a first-person perspective, incorporating kinesthetic sensations, and thus leading to a more pronounced involvement of the left premotor area compared to the other two imagery conditions. A left-sided asymmetry in kinesthetic motor imagery has been found in previous neuroimaging studies (Kuhtz-Buschbeck et al. 2003; Lorey et al. 2009; Gao et al. 2011; Orlandi et al. 2020). For example, Lorey et al. (2009) reported a greater activation over left sensorimotor and posterior parietal structures when performing a first-person perspective task involving kinesthetic motor imagery, as opposed to motor imagery trials that adopted a third-person perspective, as if they were observing another person performing the movements.

“Movement” motivational state was also associated with a large activation of the parietal ROI (see the Supplementary file), which aligns with prior literature suggesting that motor imagery is grounded in a distributed Fronto-Parietal Network enabling “emulation”. This core process would specifically deals with motor representations that generates a dynamic representation of abstract movement kinematics, supporting the internal manipulation of these representations and ensuring their short-term maintenance (Hétu et al. 2013; Ptak et al. 2017). Consistent with this notion, disruptions in this prefrontal-parietal network could explain impaired motor imagery ability (McInnes et al. 2016; Oostra et al. 2016).

In conclusion, the present study aimed at reconstructing individual patterns of neural activity associated with N400 ERP component (Proverbio and Pischedda 2023a) recorded from scalp in association with different motivational imaginary states. Indeed, ERP responses recorded in highly similar imagery paradigms were successfully classified through machine learning algorithms (Leoni et al. 2022, 2023). Here, we investigated whether it was possible to find neural signatures of mental states for brain computer interface (BCI) systems. At this aim, comparisons were made across brain activations in different ROIs identified as essential in the three motivational states. The data indicated that the temporal area, particularly over the right hemisphere, exhibited more activity during the desire for “Social Play.” The OBF\IF area was more active during listening to “Music,” and the left premotor was more active during “Movement” motivational states. These ROIs were the more distinctive markers, but all imagery conditions were associated with the activation of a common neural circuit, including the selected ROIs (especially the occipito/temporal cortex, Dijkstra et al. 2019), plus regions belonging to the default mode network, such as the medial prefrontal cortex (Frontal ROI), the posterior cingulate cortex (Limbic ROI), the precuneus and angular gyrus (Parietal ROI), and the medial temporal lobe (Temporal ROI, Grajski et al., 2019).

In a Mind Reading approach, these results hold great promise for the application of such data in BCI systems that could potentially be valuable in addressing communication challenges and improving the quality of life of patients with disorders of consciousness, such as coma or locked-in syndrome. According to some studies (Kavanagh et al. 2005) imagination would be able to activate measurable responses to visceral desires, qualitatively similar to emotional ones, regardless of whether the stimulus is perceived or imagined. Recent studies have shown how it might be possible to automatically classify brain activation signals (Leoni et al. 2021, 2022, 2023), to reveal ongoing (otherwise undetected) motivational states of patients.

## Study limits

One potential limitation of this study is the relatively small sample size; therefore, future research should aim to investigate larger samples. However, it was identified that neural markers were active in 100% of participants (see individual dipole lists in Supplementary file 1), albeit with some hemispheric differences, supporting the robustness of the data and the generalisability of the results. A further potential limitation might come from the fact that the imaginary motivational states were to be voluntary activated, and did not derive from real homeostatic needs (such as hunger or drug craving). This condition may not fully correspond to people’s experiences in real situations related to such needs, but the same criticality holds for any study involving imagery paradigms.

## Electronic Supplementary Material

Below is the link to the electronic supplementary material.

Supplementary Material 1

## Data Availability

The authors confirm that the data supporting the findings of this study are available within the article. Other information are available on request from the corresponding author. The data are not publicly available due to privacy or ethical restrictions.
