# Supplementary material for: Neural signatures of imaginary motivational states: desire for music, movement and social play
Source: Brain Topogr. 2024 Apr 16;37(5):806–25. doi: 10.1007/s10548-024-01047-1 (PMC11393278; doi:10.1007/s10548-024-01047-1)
Supplement: Supplementary file 1 — Supplementary Material 1 [file 10548_2024_1047_MOESM1_ESM.docx]

**SOCIAL PLAY**

|  | |  | | |  |  |  |  |
| --- | --- | --- | --- | --- | --- | --- | --- | --- |
| **SS01**  **MAGN.** | **T-x [mm]** | **T-y [mm]** | **T-z [mm]** | **HEM** | **LOBE** | **GYRUS** | **BA** | **ROI** |
| 3.27 | 60.6 | -2.8 | -5.9 | R | T | Middle Temporal Gyrus | 21 | TEMP |
| 3.26 | 60.6 | -21 | 35.7 | R | P | Postcentral Gyrus | 3 | PAR |
| 2.85 | 40.9 | -88.3 | 3 | R | O | Middle Occipital Gyrus | 18 | OCC |
| 2.77 | -18.5 | -91.3 | 29.7 | L | O | Cuneus | 19 | OCC |
| 2.72 | -58.5 | -4.2 | 10.7 | L | F | Precentral Gyrus | 43 | PREM |
| 2.36 | 50.8 | -66.1 | -10.9 | R | T | Fusiform Gyrus | 19 | FUSIF |
| 2.22 | -68.5 | -36.6 | -1.3 | L | T | Middle Temporal Gyrus | 21 | TEMP |
| 1.75 | -28.5 | 46.3 | -2.3 | L | F | Middle Frontal Gyrus | 10 | OBF/IF |

|  | |  | | |  |  |  |  |
| --- | --- | --- | --- | --- | --- | --- | --- | --- |
| **SS02**  **MAGN.** | **T-x [mm]** | **T-y [mm]** | **T-z [mm]** | **HEM** | **LOBE** | **GYRUS** | **BA** | **ROI** |
| 9.17 | 50.8 | -67.1 | -3.5 | R | T | Inferior Temporal Gyrus | 19 | TEMP |
| 8.40 | 40.9 | -86.4 | -12.4 | R | O | Inferior Occipital Gyrus | 18 | OCC |
| 5.68 | -58.5 | -55 | -17.6 | L | T | Fusiform Gyrus | 37 | FUSIF |
| 5.46 | 60.6 | -13.7 | 36.6 | R | F | Precentral Gyrus | 4 | PREM |
| 4.91 | 60.6 | -50.7 | 33.1 | R | P | Supramarginal Gyrus | 40 | PAR |
| 4.38 | -38.5 | -15.3 | -29.6 | L | T | Inferior Temporal Gyrus | 20 | TEMP |
| 3.59 | 50.8 | 34.3 | 14.2 | R | F | Middle Frontal Gyrus | 46 | FRONT |
| 3.31 | 21.2 | -8 | -28.9 | R | Limbic | Uncus | 36 | LIMBIC |
| 2.72 | -18.5 | -1.1 | 65 | L | F | Superior Frontal Gyrus | 6 | PREM |
| 2.34 | 11.3 | 64.4 | 16.8 | R | F | Superior Frontal Gyrus | 10 | OBF/IF |
| 1.06 | -8.5 | 57.3 | -9 | L | F | Superior Frontal Gyrus | 10 | OBF/IF |

|  | |  | | |  |  |  |  |
| --- | --- | --- | --- | --- | --- | --- | --- | --- |
| **SS03**  **MAGN.** | **T-x [mm]** | **T-y [mm]** | **T-z [mm]** | **HEM** | **LOBE** | **GYRUS** | **BA** | **ROI** |
| 3.00 | 50.8 | 33.4 | 23.1 | R | F | Middle Frontal Gyrus | 46 | FRONT |
| 2.91 | -38.5 | 10.4 | 48.1 | L | F | Middle Frontal Gyrus | 6 | PREM |
| 2.70 | 70.5 | -36.6 | -1.3 | R | T | Middle Temporal Gyrus | 21 | TEMP |
| 2.12 | -58.5 | -8.7 | -21.5 | L | T | Inferior Temporal Gyrus | 20 | TEMP |
| 2.07 | 31 | -85.4 | -19.8 | R | Cereb | Posterior Lobe,Declive |  | FUSIF |
| 2.06 | -18.5 | -8 | -28.9 | L | Limbic | Uncus | 36 | LIMBIC |
| 1.99 | -8.5 | 64.4 | 16.8 | L | F | Superior Frontal Gyrus | 10 | OBF/IF |
| 1.94 | -38.5 | -75.2 | -19.1 | L | Cereb | Posterior Lobe,Declive |  |  |
| 1.76 | 31 | -82.1 | 39.5 | R | P | Precuneus | 19 | PAR |
| 1.60 | -28.5 | -81.1 | 30.6 | L | O | Cuneus | 19 | OCC |
| 1.24 | -48.5 | -22.5 | 53.5 | L | P | Postcentral Gyrus | 1 | PAR |

|  | |  | | |  |  |  |  |
| --- | --- | --- | --- | --- | --- | --- | --- | --- |
| **SS04**  **MAGN.** | **T-x [mm]** | **T-y [mm]** | **T-z [mm]** | **HEM** | **LOBE** | **GYRUS** | **BA** | **ROI** |
| 3.43 | 31 | 20.4 | 48.9 | R | F | Superior Frontal Gyrus | 8 | FRONT |
| 2.79 | -58.5 | -55 | -17.6 | L | T | Fusiform Gyrus | 37 | FUSIF |
| 2.74 | 40.9 | -86.4 | -12.4 | R | O | Inferior Occipital Gyrus | 18 | OCC |
| 2.55 | 60.6 | -41.5 | 42.9 | R | P | Inferior Parietal Lobule | 40 | PAR |
| 2.46 | -48.5 | 8.2 | -20 | L | T | Superior Temporal Gyrus | 38 | TEMP |
| 2.46 | -48.5 | 36.3 | -3 | L | F | Middle Frontal Gyrus | 47 | OBF/IF |
| 2.30 | -8.5 | -96.5 | -13.1 | L | O | Lingual Gyrus | 17 | OCC |
| 2.20 | 60.6 | -59.9 | 23.4 | R | T | Superior Temporal Gyrus | 39 | TEMP |
| 1.59 | 31 | -15.3 | -29.6 | R | Limbic | Uncus | 20 | LIMBIC |
| 1.52 | -18.5 | -33.4 | 61.6 | L | P | Postcentral Gyrus | 3 | PAR |
| 1.46 | 1.5 | 48.2 | -17.2 | R | F | Medial Frontal Gyrus | 11 | OBF/IF |

|  | |  | | |  |  |  |  |
| --- | --- | --- | --- | --- | --- | --- | --- | --- |
| **SS05**  **MAGN.** | **T-x [mm]** | **T-y [mm]** | **T-z [mm]** | **HEM** | **LOBE** | **GYRUS** | **BA** | **ROI** |
| 9.07 | 60.6 | -55 | -17.6 | R | O | Fusiform Gyrus | 37 | FUSIF |
| 8.45 | -48.5 | -61.8 | 41.2 | L | P | Inferior Parietal Lobule | 39 | PAR |
| 6.75 | -58.5 | -55 | -17.6 | L | T | Fusiform Gyrus | 37 | FUSIF |
| 5.72 | 31 | 9.5 | 57 | R | F | Middle Frontal Gyrus | 6 | PREM |
| 5.70 | -28.5 | -15.8 | 63.3 | L | F | Precentral Gyrus | 6 | PREM |
| 5.64 | 40.9 | -81.1 | 30.6 | R | O | Superior Occipital Gyrus | 19 | OCC |
| 5.55 | -8.5 | -96.5 | -13.1 | L | O | Lingual Gyrus | 17 | OCC |
| 5.45 | 1.5 | -63.8 | 59 | R | P | Precuneus | 7 | PAR |
| 4.68 | 60.6 | 6.2 | -5.2 | R | T | Superior Temporal Gyrus | 22 | TEMP |
| 4.26 | -48.5 | 17.2 | -11.9 | L | T | Superior Temporal Gyrus | 38 | TEMP |
| 4.23 | -28.5 | -15.3 | -29.6 | L | Limbic | Uncus | 20 | LIMBIC |
| 3.68 | 1.5 | 65.3 | 7.9 | R | F | Medial Frontal Gyrus | 10 | OBF/IF |
| 2.66 | -18.5 | 52.4 | 33.7 | L | F | Superior Frontal Gyrus | 9 | FRONT |
| 2.60 | 1.5 | 52.4 | 33.7 | R | F | Medial Frontal Gyrus | 9 | FRONT |
|  |  |  |  |  |  |  |  |  |
|  |  |  |  |  |  |  |  |  |
|  | |  | | |  |  |  |  |
| **SS06**  **MAGN.** | **T-x [mm]** | **T-y [mm]** | **T-z [mm]** | **HEM** | **LOBE** | **GYRUS** | **BA** | **ROI** |
| 2.79 | -38.5 | -86.4 | -12.4 | L | O | Inferior Occipital Gyrus | 18 | OCC |
| 2.78 | -48.5 | 35.3 | 5.3 | L | F | Inferior Frontal Gyrus | 45 | OBF/IF |
| 2.41 | -38.5 | -23.2 | 62.4 | L | P | Postcentral Gyrus | 3 | PAR |
| 2.26 | -8.5 | -1.1 | 65 | L | F | Superior Frontal Gyrus | 6 | PREM |
| 1.82 | 60.6 | -13 | 27.7 | R | P | Postcentral Gyrus | 3 | PAR |
| 1.69 | 60.6 | 5.3 | 2.7 | R | T | Superior Temporal Gyrus | 22 | TEMP |
| 1.69 | -38.5 | -0.6 | -28.2 | L | T | Middle temporal Gyrus | 21 | TEMP |
| 1.59 | 50.8 | 33.4 | 23.1 | R | F | Middle Frontal Gyrus | 46 | FRONT |
| 1.28 | 40.9 | -86.4 | -12.4 | R | O | Inferior Occipital Gyrus | 18 | OCC |
| 1.19 | 60.6 | -55.9 | -10.2 | R | T | Inferior Temporal Gyrus | 37 | FUSIF |
| 0.98 | 21.2 | -0.6 | -28.2 | R | Limbic | Uncus | 36 | LIMBIC |

| **SS07**  **MAGN.** | **T-x [mm]** | **T-y [mm]** | **T-z [mm]** | **HEM** | **LOBE** | **GYRUS** | **BA** | **ROI** |
| --- | --- | --- | --- | --- | --- | --- | --- | --- |
| 6.89 | -58.5 | -55 | -17.6 | L | T | Fusiform Gyrus | 37 | FUSIF |
| 6.69 | 60.6 | 5.3 | 2.7 | R | T | Superior Temporal Gyrus | 22 | TEMP |
| 6.50 | -58.5 | 5.3 | 2.7 | L | T | Superior Temporal Gyrus | 22 | TEMP |
| 6.21 | 50.8 | 45.3 | 6.1 | R | F | Middle Frontal Gyrus | 46 | FRONT |
| 4.88 | -48.5 | 45.3 | 6.1 | L | F | Middle Frontal Gyrus | 46 | FRONT |
| 4.50 | -58.5 | -13.7 | 36.6 | L | P | Supramarginal Gyrus | 40 | PAR |
| 4.14 | -18.5 | 56.3 | -1.6 | L | F | Superior Frontal Gyrus | 10 | OBF/IF |
| 4.13 | 31 | -82.1 | 39.5 | R | P | Precuneus | 19 | PAR |
| 3.88 | 31 | -90.3 | 20.8 | R | O | Middle Occipital Gyrus | 19 | OCC |
| 3.59 | 60.6 | -13.7 | 36.6 | R | F | Precentral Gyrus | 4 | PREM |
| 1.56 | -18.5 | 19.5 | 57.8 | L | F | Superior Frontal Gyrus | 6 | PREM |

|  | |  | | |  |  |  |  |
| --- | --- | --- | --- | --- | --- | --- | --- | --- |
| **SS08**  **MAGN.** | **T-x [mm]** | **T-y [mm]** | **T-z [mm]** | **HEM** | **LOBE** | **GYRUS** | **BA** | **ROI** |
| 2.71 | 11.3 | 65.3 | 7.9 | R | F | Superior Frontal Gyrus | 10 | OBF/IF |
| 1.27 | 60.6 | -2.8 | -5.9 | R | T | Middle Temporal Gyrus | 21 | TEMP |
| 1.01 | 60.6 | -55 | -17.6 | R | O | Fusiform Gyrus | 37 | FUSIF |
| 0.89 | 31 | -89.3 | 11.9 | R | O | Middle Occipital Gyrus | 19 | OCC |
| 0.46 | -48.5 | -61.8 | 41.2 | L | P | Inferior Parietal Lobule | 39 | PAR |
| 0.45 | -68.5 | -25.5 | -8.1 | L | T | Middle temporal Gyrus | 21 | TEMP |
| 0.29 | -18.5 | -1.1 | 65 | L | F | Superior Frontal Gyrus | 6 | PREM |

|  | |  | | |  |  |  |  |
| --- | --- | --- | --- | --- | --- | --- | --- | --- |
| **SS09**  **MAGN.** | **T-x [mm]** | **T-y [mm]** | **T-z [mm]** | **HEM** | **LOBE** | **GYRUS** | **BA** | **ROI** |
| 3.51 | 31 | -53.7 | 59.8 | R | P | Superior Parietal Lobule | 7 | PAR |
| 3.09 | -38.5 | -86.4 | -12.4 | L | O | Inferior Occipital Gyrus | 18 | OCC |
| 3.04 | 60.6 | -55 | -17.6 | R | O | Fusiform Gyrus | 37 | FUSIF |
| 2.96 | 50.8 | 34.3 | 14.2 | R | F | Middle Frontal Gyrus | 46 | FRONT |
| 2.65 | 60.6 | 14.3 | 12.5 | R | F | Inferior Frontal Gyrus | 44 | OBF/IF |
| 2.58 | -38.5 | -62.8 | 50.1 | L | P | Superior Parietal Lobule | 7 | PAR |
| 2.40 | 60.6 | 6.2 | -5.2 | R | T | Superior Temporal Gyrus | 22 | TEMP |
| 2.37 | 31 | -90.3 | 20.8 | R | O | Middle Occipital Gyrus | 19 | OCC |
| 1.37 | -18.5 | -8 | -28.9 | L | Limbic | Uncus | 36 | LIMBIC |
| 1.00 | -38.5 | 32.4 | 32 | L | F | Middle Frontal Gyrus | 9 | FRONT |
| 0.96 | -58.5 | 13.3 | 21.4 | L | F | Inferior Frontal Gyrus | 45 | OBF/IF |
| 0.93 | -38.5 | -0.4 | 56.1 | L | F | Middle Frontal Gyrus | 6 | PREM |
| 0.83 | 11.3 | 35.3 | 5.3 | R | Limbic | Anterior Cingulate | 24 | LIMBIC |
| 0.71 | 1.5 | 19.5 | 57.8 | R | F | Superior Frontal Gyrus | 6 | PREM |

|  | |  | | |  |  |  |  |
| --- | --- | --- | --- | --- | --- | --- | --- | --- |
| **SS10**  **MAGN.** | **T-x [mm]** | **T-y [mm]** | **T-z [mm]** | **HEM** | **LOBE** | **GYRUS** | **BA** | **ROI** |
| 3.62 | 40.9 | -87.3 | -4.9 | R | O | Inferior Occipital Gyrus | 18 | OCC |
| 3.62 | -28.5 | -15.8 | 63.3 | L | F | Precentral Gyrus | 6 | PREM |
| 3.28 | 60.6 | -44.8 | -16.9 | R | T | Inferior Temporal Gyrus | 20 | TEMP |
| 2.86 | -58.5 | -55 | -17.6 | L | T | Fusiform Gyrus | 37 | FUSIF |
| 2.75 | 60.6 | -29.4 | 26 | R | P | Inferior Parietal Lobule | 40 | PAR |
| 2.30 | -48.5 | 22.4 | 31.1 | L | F | Middle Frontal Gyrus | 9 | FRONT |
| 2.12 | -8.5 | -63.8 | 59 | L | P | Superior Parietal Lobule | 7 | PAR |
| 1.86 | -58.5 | -8.7 | -21.5 | L | T | Inferior Temporal Gyrus | 20 | TEMP |
| 1.83 | -18.5 | -8 | -28.9 | L | Limbic | Uncus | 36 | LIMBIC |
| 1.23 | 50.8 | 22.4 | 31.1 | R | F | Middle Frontal Gyrus | 9 | FRONT |

|  | |  | | |  |  |  |  |
| --- | --- | --- | --- | --- | --- | --- | --- | --- |
| **SS11**  **MAGN.** | **T-x [mm]** | **T-y [mm]** | **T-z [mm]** | **HEM** | **LOBE** | **GYRUS** | **BA** | **ROI** |
| 4.94 | -48.5 | -33.7 | -23.6 | L | T | Fusiform Gyrus | 20 | FUSIF |
| 4.60 | 60.6 | -3.5 | 1.8 | R | T | Superior Temporal Gyrus | 22 | TEMP |
| 4.53 | -58.5 | -8.7 | -21.5 | L | T | Inferior Temporal Gyrus | 20 | TEMP |
| 4.08 | -18.5 | -90.3 | 20.8 | L | O | Cuneus | 18 | OCC |
| 4.05 | 40.9 | -81.1 | 30.6 | R | O | Superior Occipital Gyrus | 19 | OCC |
| 3.83 | 11.3 | 65.3 | 7.9 | R | F | Superior Frontal Gyrus | 10 | OBF/IF |
| 3.82 | 40.9 | -7.8 | 55.2 | R | F | Precentral Gyrus | 6 | PREM |
| 2.17 | -8.5 | -63.8 | 59 | L | P | Superior Parietal Lobule | 7 | PAR |
| 1.91 | 21.2 | 9.1 | -27.5 | R | Limbic | Uncus | 38 | LIMBIC |
| 1.50 | -18.5 | 30.5 | 49.8 | L | F | Superior Frontal Gyrus | 8 | FRONT |

|  | |  | | |  |  |  |  |
| --- | --- | --- | --- | --- | --- | --- | --- | --- |
| **SS12**  **MAGN.** | **T-x [mm]** | **T-y [mm]** | **T-z [mm]** | **HEM** | **LOBE** | **GYRUS** | **BA** | **ROI** |
| 7.70 | -48.5 | -0.6 | -28.2 | L | T | Inferior Temporal Gyrus | 20 | TEMP |
| 6.68 | 50.8 | 8.2 | -20 | R | T | Superior Temporal Gyrus | 38 | TEMP |
| 6.50 | 40.9 | 27.2 | -11.2 | R | F | Inferior Frontal Gyrus | 47 | OBF/IF |
| 6.24 | 40.9 | 45.3 | 6.1 | R | F | Middle Frontal Gyrus | 46 | FRONT |
| 6.09 | -58.5 | -55 | -17.6 | L | T | Fusiform Gyrus | 37 | FUSIF |
| 5.25 | 60.6 | -55 | -17.6 | R | O | Fusiform Gyrus | 37 | FUSIF |
| 4.78 | -38.5 | -86.4 | -12.4 | L | O | Inferior Occipital Gyrus | 18 | OCC |
| 4.36 | 31 | -82.1 | 39.5 | R | P | Precuneus | 19 | PAR |
| 2.13 | 31 | -23.2 | 62.4 | R | F | Precentral Gyrus | 4 | PREM |

|  | |  | | |  |  |  |  |
| --- | --- | --- | --- | --- | --- | --- | --- | --- |
| **SS13**  **MAGN.** | **T-x [mm]** | **T-y [mm]** | **T-z [mm]** | **HEM** | **LOBE** | **GYRUS** | **BA** | **ROI** |
| 3.86 | 11.3 | 65.3 | 7.9 | R | F | Superior Frontal Gyrus | 10 | OBF/IF |
| 2.78 | 60.6 | -55.9 | -10.2 | R | T | Inferior Temporal Gyrus | 37 | FUSIF |
| 2.59 | 31 | -97.5 | -5.7 | R | O | Inferior Occipital Gyrus | 18 | OCC |
| 1.88 | -68.5 | -25.5 | -8.1 | L | T | Middle temporal Gyrus | 21 | TEMP |
| 1.70 | 50.8 | -0.6 | -28.2 | R | T | Middle Temporal Gyrus | 21 | TEMP |
| 1.52 | -48.5 | 11.4 | 39.2 | L | F | Middle Frontal Gyrus | 8 | FRONT |
| 1.49 | 1.5 | -73 | 49.2 | R | P | Precuneus | 7 | PAR |
| 1.32 | -8.5 | -0.6 | -28.2 | L | Limbic | Uncus | 28 | LIMBIC |
| 1.29 | 11.3 | 12.4 | 30.3 | R | Limbic | Cingulate Gyrus | 24 | LIMBIC |
| 1.13 | -28.5 | -97.5 | -5.7 | L | O | Lingual Gyrus | 18 | OCC |
| 1.12 | 40.9 | 2.4 | 29.4 | R | F | Precentral Gyrus | 6 | PREM |
| 1.00 | -28.5 | -15.8 | 63.3 | L | F | Precentral Gyrus | 6 | PREM |

|  | |  | | |  |  |  |  |
| --- | --- | --- | --- | --- | --- | --- | --- | --- |
| **SS14**  **MAGN.** | **T-x [mm]** | **T-y [mm]** | **T-z [mm]** | **HEM** | **LOBE** | **GYRUS** | **BA** | **ROI** |
| 2.71 | -58.5 | 4.3 | 11.6 | L | F | Precentral Gyrus | 6 | PREM |
| 2.21 | -48.5 | 36.3 | -3 | L | F | Middle Frontal Gyrus | 47 | OBF/IF |
| 2.01 | 50.8 | -22.5 | 53.5 | R | P | Postcentral Gyrus | 1 | PAR |
| 1.78 | 60.6 | -2.1 | -13.3 | R | Limbic | Uncus | 20 | LIMBIC |
| 1.77 | -68.5 | -36.6 | -1.3 | L | T | Middle Temporal Gyrus | 21 | TEMP |
| 1.69 | -58.5 | -55.9 | -10.2 | L | T | Inferior Temporal Gyrus | 37 | FUSIF |
| 1.61 | -48.5 | -77.2 | -4.2 | L | O | Middle Occipital Gyrus | 19 | OCC |
| 1.41 | 40.9 | -88.3 | 3 | R | O | Middle Occipital Gyrus | 18 | OCC |
| 1.34 | 1.5 | 8.5 | 65.9 | R | F | Superior Frontal Gyrus | 6 | PREM |
| 1.33 | -8.5 | -63.8 | 59 | L | P | Superior Parietal Lobule | 7 | PAR |
| 1.09 | -18.5 | 41.4 | 41.8 | L | F | Superior Frontal Gyrus | 8 | FRONT |
| 0.96 | 50.8 | 45.3 | 6.1 | R | F | Middle Frontal Gyrus | 46 | FRONT |

|  | |  | | |  |  |  |  |
| --- | --- | --- | --- | --- | --- | --- | --- | --- |
| **SS15**  **MAGN.** | **T-x [mm]** | **T-y [mm]** | **T-z [mm]** | **HEM** | **LOBE** | **GYRUS** | **BA** | **ROI** |
| 4.61 | 50.8 | 33.4 | 23.1 | R | F | Middle Frontal Gyrus | 46 | FRONT |
| 3.82 | 31 | -15.8 | 63.3 | R | F | Precentral Gyrus | 6 | PREM |
| 2.42 | 40.9 | -88.3 | 3 | R | O | Middle Occipital Gyrus | 18 | OCC |
| 2.18 | 60.6 | -55 | -17.6 | R | O | Fusiform Gyrus | 37 | FUSIF |
| 1.98 | 70.5 | -17.5 | -7.3 | R | T | Middle temporal Gyrus | 21 | TEMP |
| 1.81 | -28.5 | -53.7 | 59.8 | L | P | Superior Parietal Lobule | 7 | PAR |
| 1.74 | -58.5 | -45.8 | -9.5 | L | T | Middle Temporal Gyrus | 37 | FUSIF |
| 1.71 | -58.5 | -58.9 | 14.5 | L | T | Superior Temporal Gyrus | 22 | TEMP |
| 1.59 | -18.5 | -91.3 | 29.7 | L | O | Cuneus | 19 | OCC |
| 1.31 | 60.6 | -41.5 | 42.9 | R | P | Inferior Parietal Lobule | 40 | PAR |
| 0.95 | -58.5 | 13.3 | 21.4 | L | F | Inferior Frontal Gyrus | 45 | OBF/IF |
| 0.95 | -48.5 | 33.4 | 23.1 | L | F | Middle Frontal Gyrus | 46 | FRONT |

|  | |  | | |  |  |  |  |
| --- | --- | --- | --- | --- | --- | --- | --- | --- |
| **SS16**  **MAGN.** | **T-x [mm]** | **T-y [mm]** | **T-z [mm]** | **HEM** | **LOBE** | **GYRUS** | **BA** | **ROI** |
| 4.21 | -58.5 | -9.4 | -14 | L | T | Inferior Temporal Gyrus | 21 | TEMP |
| 2.72 | 21.2 | 9.1 | -27.5 | R | Limbic | Uncus | 38 | LIMBIC |
| 1.94 | 40.9 | -0.4 | 56.1 | R | F | Middle Frontal Gyrus | 6 | PREM |
| 1.89 | -58.5 | 2.4 | 29.4 | L | F | Precentral Gyrus | 6 | PREM |
| 1.82 | 40.9 | -86.4 | -12.4 | R | O | Inferior Occipital Gyrus | 18 | OCC |
| 1.81 | -28.5 | -90.3 | 20.8 | L | O | Middle Occipital Gyrus | 19 | OCC |
| 1.78 | 60.6 | 5.3 | 2.7 | R | T | Superior Temporal Gyrus | 22 | TEMP |
| 1.68 | 40.9 | -55 | -17.6 | R | O | Fusiform Gyrus | 37 | FUSIF |
| 1.47 | 21.2 | 52.4 | 33.7 | R | F | Superior Frontal Gyrus | 9 | FRONT |
| 1.02 | 11.3 | -63.8 | 59 | R | P | Superior Parietal Lobule | 7 | PAR |

|  | |  | | |  |  |  |  |
| --- | --- | --- | --- | --- | --- | --- | --- | --- |
| **SS17**  **MAGN.** | **T-x [mm]** | **T-y [mm]** | **T-z [mm]** | **HEM** | **LOBE** | **GYRUS** | **BA** | **ROI** |
| 3.60 | 40.9 | -80.1 | 21.7 | R | T | Middle Temporal Gyrus | 19 | TEMP |
| 3.49 | 40.9 | -88.3 | 3 | R | O | Middle Occipital Gyrus | 18 | OCC |
| 1.98 | -28.5 | -97.5 | -5.7 | L | O | Lingual Gyrus | 18 | OCC |
| 1.95 | -28.5 | -15.8 | 63.3 | L | F | Precentral Gyrus | 6 | PREM |
| 1.44 | -58.5 | -8.7 | -21.5 | L | T | Inferior Temporal Gyrus | 20 | TEMP |
| 1.40 | -48.5 | -33.7 | -23.6 | L | T | Fusiform Gyrus | 20 | FUSIF |
| 1.27 | -18.5 | -8 | -28.9 | L | Limbic | Uncus | 36 | LIMBIC |
| 1.22 | -8.5 | -63.8 | 59 | L | P | Superior Parietal Lobule | 7 | PAR |
| 1.13 | 50.8 | 33.4 | 23.1 | R | F | Middle Frontal Gyrus | 46 | FRONT |
| 1.01 | 60.6 | -6.3 | 37.4 | R | F | Precentral Gyrus | 6 | PREM |
| 0.73 | 11.3 | 64.4 | 16.8 | R | F | Superior Frontal Gyrus | 10 | OBF/IF |
| 0.43 | -48.5 | 33.4 | 23.1 | L | F | Middle Frontal Gyrus | 46 | FRONT |

|  | |  | | |  |  |  |  |
| --- | --- | --- | --- | --- | --- | --- | --- | --- |
| **SS18**  **MAGN.** | **T-x [mm]** | **T-y [mm]** | **T-z [mm]** | **HEM** | **LOBE** | **GYRUS** | **BA** | **ROI** |
| 8.52 | -18.5 | 52.4 | 33.7 | L | F | Superior Frontal Gyrus | 9 | FRONT |
| 4.67 | -58.5 | -46.8 | -2.1 | L | T | Middle Temporal Gyrus | 21 | TEMP |
| 3.24 | 1.5 | -98.5 | 2.1 | R | O | Cuneus | 18 | OCC |
| 3.10 | 31 | -15.8 | 63.3 | R | F | Precentral Gyrus | 6 | PREM |
| 2.99 | 21.2 | -24.5 | -15.5 | R | Limbic | Parahippocampal Gyrus | 35 | LIMBIC |
| 2.54 | 60.6 | -16.8 | -14.8 | R | T | Inferior Temporal Gyrus | 20 | TEMP |
| 2.39 | 40.9 | 55.3 | 7 | R | F | Middle Frontal Gyrus | 10 | OBF/IF |

*Tables of active electromagnetic dipoles in swLORETA for the N400 (400-600 ms) component, obtained in the eighteen participants included in the ANOVA analysis, measured during the “Social Play” motivational state. MAGN. = magnitude in nA.*

**MUSIC**

|  | |  | | |  |  |  |  |
| --- | --- | --- | --- | --- | --- | --- | --- | --- |
| **SS01**  **MAGN.** | **T-x [mm]** | **T-y [mm]** | **T-z [mm]** | **HEM** | **LOBE** | **GYRUS** | **BA** | **ROI** |
| 7.02 | 40.9 | -86.4 | -12.4 | R | O | Inferior Occipital Gyrus | 18 | OCC |
| 6.94 | 11.3 | 65.3 | 7.9 | R | F | Superior Frontal Gyrus | 10 | OBF/IF |
| 6.90 | 60.6 | -55.9 | -10.2 | R | T | Inferior Temporal Gyrus | 37 | FUSIF |
| 6.37 | 50.8 | -0.6 | -28.2 | R | T | Middle temporal gyrus | 21 | TEMP |
| 3.81 | -8.5 | -0.6 | -28.2 | L | Limbic | Uncus | 28 | LIMBIC |
| 3.02 | -18.5 | -91.3 | 29.7 | L | O | Cuneus | 19 | OCC |
| 2.76 | -68.5 | -36.6 | -1.3 | L | T | Middle temporal gyrus | 21 | TEMP |
| 2.47 | -18.5 | 41.4 | 41.8 | L | F | Superior Frontal Gyrus | 8 | FRONT |
| 1.80 | 1.5 | -20.3 | 26.8 | R | Limbic | Cingulate Gyrus | 31 | LIMBIC |
| 1.57 | -58.5 | -31.4 | 43.8 | L | P | Inferior PAR Lobule | 40 | PAR |
| 1.52 | 31.0 | -15.8 | 63.3 | R | F | Precentral Gyrus | 6 | PREM |
| 1.36 | -58.5 | 2.4 | 29.4 | L | F | Precentral Gyrus | 6 | PREM |
| 1.34 | 21.2 | 30.5 | 49.8 | R | F | Superior Frontal Gyrus | 8 | FRONT |

|  | |  | | |  |  |  |  |
| --- | --- | --- | --- | --- | --- | --- | --- | --- |
| **SS02**  **MAGN.** | **T-x [mm]** | **T-y [mm]** | **T-z [mm]** | **HEM** | **LOBE** | **GYRUS** | **BA** | **ROI** |
| 6.80 | 60.6 | -2.8 | -5.9 | R | T | Middle Temporal Gyrus | 21 | TEMP |
| 6.05 | 40.9 | -86.4 | -12.4 | R | O | Inferior Occipital Gyrus | 18 | OCC |
| 4.05 | -58.5 | -13 | 27.7 | L | P | Postcentral Gyrus | 3 | PAR |
| 3.51 | -58.5 | -55 | -17.6 | L | T | Fusiform Gyrus | 37 | FUSIF |
| 3.38 | -58.5 | -47.8 | 6.4 | L | T | Middle Temporal Gyrus | 21 | TEMP |
| 3.18 | -48.5 | 11.4 | 39.2 | L | F | Middle Frontal Gyrus | 8 | FRONT |
| 2.62 | 11.3 | 29.5 | 58.7 | R | F | Superior Frontal Gyrus | 6 | PREM |
| 2.04 | -8.5 | -91.3 | 29.7 | L | O | Cuneus | 19 | OCC |
| 1.77 | 11.3 | 35.3 | 5.3 | R | Limbic | Anterior Cingulate | 24 | LIMBIC |
|  |  |  |  |  |  |  |  |  |
|  |  |  |  |  |  |  |  |  |
|  | |  | | |  |  |  |  |
| **SS03**  **MAGN.** | **T-x [mm]** | **T-y [mm]** | **T-z [mm]** | **HEM** | **LOBE** | **GYRUS** | **BA** | **ROI** |
| 8.74 | 40.9 | -86.4 | -12.4 | R | O | Inferior Occipital Gyrus | 18 | OCC |
| 8.73 | 50.8 | -66.1 | -10.9 | R | T | Fusiform Gyrus | 19 | FUSIF |
| 4.32 | 60.6 | -39.6 | 25.1 | R | P | Inferior PAR Lobule | 40 | PAR |
| 3.23 | 60.6 | 5.3 | 2.7 | R | T | Superior Temporal Gyrus | 22 | TEMP |
| 2.99 | 50.8 | 36.3 | -3 | R | F | Inferior Frontal Gyrus | 47 | OBF/IF |
| 2.70 | -58.5 | -55 | -17.6 | L | T | Fusiform Gyrus | 37 | FUSIF |
| 2.59 | -18.5 | -8 | -28.9 | L | Limbic | Uncus | 36 | LIMBIC |
| 2.17 | 1.5 | 29.5 | 58.7 | R | F | Superior Frontal Gyrus | 6 | PREM |
| 1.94 | 21.2 | 41.4 | 41.8 | R | F | Superior Frontal Gyrus | 8 | FRONT |
| 1.88 | -58.5 | -58.9 | 14.5 | L | T | Superior Temporal Gyrus | 22 | TEMP |
| 1.81 | -28.5 | -88.3 | 3 | L | O | Middle Occipital Gyrus | 18 | OCC |
| 1.75 | -38.5 | -72 | 40.3 | L | P | Precuneus | 19 | PAR |
| 0.96 | -28.5 | 55.3 | 7 | L | F | Middle Frontal Gyrus | 10 | OBF/IF |
|  |  |  |  |  |  |  |  |  |
|  |  |  |  |  |  |  |  |  |

|  | |  | | |  |  |  |  |
| --- | --- | --- | --- | --- | --- | --- | --- | --- |
| **SS04**  **MAGN.** | **T-x [mm]** | **T-y [mm]** | **T-z [mm]** | **HEM** | **LOBE** | **GYRUS** | **BA** | **ROI** |
| 4.40 | 60.6 | -55 | -17.6 | R | O | Fusiform Gyrus | 37 | FUSIF |
| 4.35 | 11.3 | 65.3 | 7.9 | R | F | Superior Frontal Gyrus | 10 | OBF/IF |
| 4.22 | 31 | -97.5 | -5.7 | R | O | Inferior Occipital Gyrus | 18 | OCC |
| 3.70 | 60.6 | -30.4 | 34.9 | R | P | Inferior PAR Lobule | 40 | PAR |
| 3.50 | -28.5 | -15.8 | 63.3 | L | F | Precentral Gyrus | 6 | PREM |
| 3.15 | -58.5 | -9.4 | -14 | L | T | Inferior Temporal Gyrus | 21 | TEMP |
| 2.71 | -58.5 | 14.3 | 12.5 | L | F | Inferior Frontal Gyrus | 44 | OBF/IF |
| 2.70 | -18.5 | -45.8 | -9.5 | L | Cereb | Anterior Lobe,Culmen | 19 | FUSIF |
| 2.60 | -58.5 | -50.7 | 33.1 | L | P | Supramarginal Gyrus | 40 | PAR |
| 2.46 | -48.5 | 34.3 | 14.2 | L | F | Middle Frontal Gyrus | 46 | FRONT |
| 2.31 | 50.8 | 33.4 | 23.1 | R | F | Middle Frontal Gyrus | 46 | FRONT |
| 1.89 | 11.3 | -9.4 | -14 | R | Limbic | Parahippocampal Gyrus | 34 | LIMBIC |
| 1.87 | 50.8 | 8.2 | -20 | R | T | Superior Temporal Gyrus | 38 | TEMP |
| 1.56 | 21.2 | -15.8 | 63.3 | R | F | Precentral Gyrus | 6 | PREM |
|  |  |  |  |  |  |  |  |  |
|  |  |  |  |  |  |  |  |  |
|  | |  | | |  |  |  |  |
| **SS05**  **MAGN.** | **T-x [mm]** | **T-y [mm]** | **T-z [mm]** | **HEM** | **LOBE** | **GYRUS** | **BA** | **ROI** |
| 2.63 | -58.5 | -44.8 | -16.9 | L | T | Inferior Temporal Gyrus | 20 | TEMP |
| 2.12 | 1.5 | 47.3 | -9.7 | R | F | Medial Frontal Gyrus | 10 | OBF/IF |
| 2.08 | 50.8 | 22.4 | 31.1 | R | F | Middle Frontal Gyrus | 9 | FRONT |
| 2.05 | -18.5 | 55.3 | 7 | L | F | Superior Frontal Gyrus | 10 | OBF/IF |
| 1.87 | 60.6 | -57.9 | 5.6 | R | T | Middle Temporal Gyrus | 21 | TEMP |
| 1.62 | -8.5 | -99.4 | 11 | L | O | Cuneus | 18 | OCC |
| 1.57 | -48.5 | -71 | 31.4 | L | P | Angular Gyrus | 39 | PAR |
| 1.55 | -48.5 | 0.4 | 47.2 | L | F | Precentral Gyrus | 6 | PREM |
| 1.46 | 21.2 | -1.1 | 65 | R | F | Superior Frontal Gyrus | 6 | PREM |
| 1.16 | 31 | -53.7 | 59.8 | R | P | Superior PAR Lobule | 7 | PAR |
|  |  |  |  |  |  |  |  |  |
|  |  |  |  |  |  |  |  |  |
|  | |  | | |  |  |  |  |
| **SS06**  **MAGN.** | **T-x [mm]** | **T-y [mm]** | **T-z [mm]** | **HEM** | **LOBE** | **GYRUS** | **BA** | **ROI** |
| 3.67 | 40.9 | -86.4 | -12.4 | R | O | Inferior Occipital Gyrus | 18 | OCC |
| 3.59 | 50.8 | -66.1 | -10.9 | R | T | Fusiform Gyrus | 19 | FUSIF |
| 3.17 | -48.5 | 35.3 | 5.3 | L | F | Inferior Frontal Gyrus | 45 | OBF/IF |
| 2.87 | 31 | -15.8 | 63.3 | R | F | Precentral Gyrus | 6 | PREM |
| 2.86 | 60.6 | -41.5 | 42.9 | R | P | Inferior PAR Lobule | 40 | PAR |
| 2.77 | -58.5 | -55 | -17.6 | L | T | Fusiform Gyrus | 37 | FUSIF |
| 2.16 | 21.2 | 30.5 | 49.8 | R | F | Superior Frontal Gyrus | 8 | FRONT |
| 2.11 | -58.5 | -1.4 | -20.8 | L | T | Middle temporal Gyrus | 21 | TEMP |
| 1.74 | -8.5 | -96.5 | -13.1 | L | O | Lingual Gyrus | 17 | OCC |
| 1.36 | -38.5 | -72 | 40.3 | L | P | Precuneus | 19 | PAR |
| 1.33 | 50.8 | -0.6 | -28.2 | R | T | Middle Temporal Gyrus | 21 | TEMP |
| 1.17 | 40.9 | 55.3 | 7 | R | F | Middle Frontal Gyrus | 10 | OBF/IF |

|  | |  | | |  |  |  |  |
| --- | --- | --- | --- | --- | --- | --- | --- | --- |
| **SS07**  **MAGN.** | **T-x [mm]** | **T-y [mm]** | **T-z [mm]** | **HEM** | **LOBE** | **GYRUS** | **BA** | **ROI** |
| 6.41 | 50.8 | -66.1 | -10.9 | R | T | Fusiform Gyrus | 19 | FUSIF |
| 6.34 | 40.9 | -86.4 | -12.4 | R | O | Inferior Occipital Gyrus | 18 | OCC |
| 4.41 | -28.5 | 53.4 | 24.8 | L | F | Superior Frontal Gyrus | 10 | OBF/IF |
| 4.07 | -28.5 | -8.5 | 64.2 | L | F | Superior Frontal Gyrus | 6 | PREM |
| 3.67 | 1.5 | -73 | 49.2 | R | P | Precuneus | 7 | PAR |
| 3.66 | 40.9 | 55.3 | 7 | R | F | Middle Frontal Gyrus | 10 | OBF/IF |
| 3.50 | -48.5 | 22.4 | 31.1 | L | F | Middle Frontal Gyrus | 9 | FRONT |
| 3.46 | 40.9 | 18.2 | -19.3 | R | T | Superior Temporal Gyrus | 38 | TEMP |
| 3.32 | -48.5 | -78.2 | 3.8 | L | O | Middle Occipital Gyrus | 19 | OCC |
| 2.98 | -8.5 | -0.6 | -28.2 | L | Limbic | Uncus | 28 | LIMBIC |
| 2.12 | 1.5 | 40.5 | 50.7 | R | F | Superior Frontal Gyrus | 8 | FRONT |
| 2.09 | -68.5 | -18.2 | 0.1 | L | T | Superior Temporal Gyrus | 22 | TEMP |
| 1.50 | 21.2 | -8.5 | 64.2 | R | F | Medial Frontal Gyrus | 6 | PREM |
|  |  |  |  |  |  |  |  |  |
|  |  |  |  |  |  |  |  |  |
|  | |  | | |  |  |  |  |
| **SS08**  **MAGN.** | **T-x [mm]** | **T-y [mm]** | **T-z [mm]** | **HEM** | **LOBE** | **GYRUS** | **BA** | **ROI** |
| 5.19 | 21.2 | -96.5 | -13.1 | R | O | Lingual Gyrus | 18 | OCC |
| 4.35 | -48.5 | 0.4 | 47.2 | L | F | Precentral Gyrus | 6 | PREM |
| 4.07 | -28.5 | -33.4 | 61.6 | L | P | Postcentral Gyrus | 3 | PAR |
| 3.81 | 11.3 | 40.5 | 50.7 | R | F | Superior Frontal Gyrus | 8 | FRONT |
| 3.76 | 1.5 | 8.5 | 65.9 | R | F | Superior Frontal Gyrus | 6 | PREM |
| 3.69 | 60.6 | -55.9 | -10.2 | R | T | Inferior Temporal Gyrus | 37 | FUSIF |
| 2.58 | -38.5 | 46.3 | -2.3 | L | F | Inferior Frontal Gyrus | 10 | OBF/IF |
| 2.50 | -68.5 | -36.6 | -1.3 | L | T | Middle Temporal Gyrus | 21 | TEMP |
| 2.46 | -58.5 | -56.9 | -2.8 | L | T | Inferior Temporal Gyrus | 37 | FUSIF |
| 1.63 | 21.2 | -0.6 | -28.2 | R | Limbic | Uncus | 36 | LIMBIC |
| 1.61 | 50.8 | -0.6 | -28.2 | R | T | Middle Temporal Gyrus | 21 | TEMP |
| 1.56 | -8.5 | -0.6 | -28.2 | L | Limbic | Uncus | 28 | LIMBIC |
|  |  |  |  |  |  |  |  |  |
|  | |  | | |  |  |  |  |
| **SS09**  **MAGN.** | **T-x [mm]** | **T-y [mm]** | **T-z [mm]** | **HEM** | **LOBE** | **GYRUS** | **BA** | **ROI** |
| 5.45 | -48.5 | -77.2 | -4.2 | L | O | Middle Occipital Gyrus | 19 | OCC |
| 5.37 | 60.6 | -55 | -17.6 | R | O | Fusiform Gyrus | 37 | FUSIF |
| 4.80 | -58.5 | 5.3 | 2.7 | L | T | Superior Temporal Gyrus | 22 | TEMP |
| 4.55 | -58.5 | -56.9 | -2.8 | L | T | Inferior Temporal Gyrus | 37 | FUSIF |
| 4.28 | -48.5 | 26.3 | -3.7 | L | F | Inferior Frontal Gyrus | 47 | OBF/IF |
| 4.24 | -28.5 | -15.8 | 63.3 | L | F | Precentral Gyrus | 6 | PREM |
| 4.17 | 40.9 | -72 | 40.3 | R | P | Precuneus | 19 | PAR |
| 3.73 | 50.8 | 45.3 | 6.1 | R | F | Middle Frontal Gyrus | 46 | FRONT |
| 3.59 | 40.9 | -88.3 | 3 | R | O | Middle Occipital Gyrus | 18 | OCC |
| 3.40 | 60.6 | 4.3 | 11.6 | R | F | Precentral Gyrus | 6 | PREM |
| 2.41 | 50.8 | -0.6 | -28.2 | R | T | Middle Temporal Gyrus | 21 | TEMP |
| 2.31 | -48.5 | -71 | 31.4 | L | P | Angular Gyrus | 39 | PAR |
| 1.96 | -8.5 | -0.6 | -28.2 | L | Limbic | Uncus | 28 | LIMBIC |

|  | |  | | |  |  |  |  |
| --- | --- | --- | --- | --- | --- | --- | --- | --- |
| **SS10**  **MAGN.** | **T-x [mm]** | **T-y [mm]** | **T-z [mm]** | **HEM** | **LOBE** | **GYRUS** | **BA** | **ROI** |
| 3.34 | 50.8 | -33.7 | -23.6 | R | T | Fusiform Gyrus | 20 | FUSIF |
| 3.32 | -18.5 | -8 | -28.9 | L | Limbic | Uncus | 36 | LIMBIC |
| 3.27 | -58.5 | -8.7 | -21.5 | L | T | Inferior Temporal Gyrus | 20 | TEMP |
| 3.21 | -48.5 | -33.7 | -23.6 | L | T | Fusiform Gyrus | 20 | FUSIF |
| 3.00 | -48.5 | 45.3 | 6.1 | L | F | Middle Frontal Gyrus | 46 | FRONT |
| 2.95 | 21.2 | 9.1 | -27.5 | R | Limbic | Uncus | 38 | LIMBIC |
| 2.76 | 50.8 | -0.6 | -28.2 | R | T | Middle Temporal Gyrus | 21 | TEMP |
| 2.19 | 60.6 | -13.7 | 36.6 | R | F | Precentral Gyrus | 4 | PREM |
| 2.11 | -8.5 | -99.4 | 11 | L | O | Cuneus | 18 | OCC |
| 1.96 | 50.8 | -42.5 | 51.8 | R | P | Inferior PAR Lobule | 40 | PAR |
| 1.76 | 40.9 | -79.2 | 12.7 | R | O | Middle Occipital Gyrus | 19 | OCC |
| 1.46 | -48.5 | -71 | 31.4 | L | P | Angular Gyrus | 39 | PAR |
| 1.34 | 60.6 | 13.3 | 21.4 | R | F | Inferior Frontal Gyrus | 45 | OBF/IF |
|  |  |  |  |  |  |  |  |  |
|  |  |  |  |  |  |  |  |  |
|  | |  | | |  |  |  |  |
| **SS11**  **MAGN.** | **T-x [mm]** | **T-y [mm]** | **T-z [mm]** | **HEM** | **LOBE** | **GYRUS** | **BA** | **ROI** |
| 6.38 | 11.3 | 65.3 | 7.9 | R | F | Superior Frontal Gyrus | 10 | OBF/IF |
| 5.91 | -28.5 | 55.3 | 7 | L | F | Middle Frontal Gyrus | 10 | OBF/IF |
| 1.76 | 31 | -8.5 | 64.2 | R | F | Precentral Gyrus | 6 | PREM |
| 1.49 | -8.5 | -63.8 | 59 | L | P | Superior PAR Lobule | 7 | PAR |
| 1.41 | 31 | -97.5 | -5.7 | R | O | Inferior Occipital Gyrus | 18 | OCC |
| 1.19 | 70.5 | -26.5 | -0.6 | R | T | Middle Temporal Gyrus | 21 | TEMP |
| 1.11 | 40.9 | -55 | -17.6 | R | O | Fusiform Gyrus | 37 | FUSIF |
| 1.10 | 21.2 | -16.1 | -22.2 | R | Limbic | Parahippocampal Gyrus | 28 | LIMBIC |
| 1.10 | -58.5 | -55 | -17.6 | L | T | Fusiform Gyrus | 37 | FUSIF |
|  |  |  |  |  |  |  |  |  |
|  |  |  |  |  |  |  |  |  |
|  | |  | | |  |  |  |  |
| **SS12**  **MAGN.** | **T-x [mm]** | **T-y [mm]** | **T-z [mm]** | **HEM** | **LOBE** | **GYRUS** | **BA** | **ROI** |
| 2.67 | 31 | -15.8 | 63.3 | R | F | Precentral Gyrus | 6 | PREM |
| 2.03 | -58.5 | -55 | -17.6 | L | T | Fusiform Gyrus | 37 | FUSIF |
| 1.37 | -58.5 | -8.7 | -21.5 | L | T | Inferior Temporal Gyrus | 20 | TEMP |
| 1.34 | -18.5 | -91.3 | 29.7 | L | O | Cuneus | 19 | OCC |
| 1.31 | 40.9 | -81.1 | 30.6 | R | O | Superior Occipital Gyrus | 19 | OCC |
| 0.97 | -18.5 | 30.5 | 49.8 | L | F | Superior Frontal Gyrus | 8 | FRONT |
| 0.96 | -48.5 | 0.4 | 47.2 | L | F | Precentral Gyrus | 6 | PREM |
| 0.94 | 60.6 | -55.9 | -10.2 | R | T | Inferior Temporal Gyrus | 37 | FUSIF |
| 0.89 | 1.5 | 40.5 | 50.7 | R | F | Superior Frontal Gyrus | 8 | FRONT |
| 0.78 | 11.3 | -9.4 | -14 | R | Limbic | Parahippocampal Gyrus | 34 | LIMBIC |
| 0.73 | 1.5 | 18.2 | -19.3 | R | F | Rectal Gyrus | 11 | OBF/IF |
| 0.61 | -48.5 | -42.5 | 51.8 | L | P | Inferior PAR Lobule | 40 | PAR |

|  | |  | | |  |  |  |  |
| --- | --- | --- | --- | --- | --- | --- | --- | --- |
| **SS13**  **MAGN.** | **T-x [mm]** | **T-y [mm]** | **T-z [mm]** | **HEM** | **LOBE** | **GYRUS** | **BA** | **ROI** |
| 3.93 | 60.6 | -55 | -17.6 | R | O | Fusiform Gyrus | 37 | FUSIF |
| 3.73 | 31 | -90.3 | 20.8 | R | O | Middle Occipital Gyrus | 19 | OCC |
| 3.66 | 50.8 | -33.7 | -23.6 | R | T | Fusiform Gyrus | 20 | FUSIF |
| 3.41 | -38.5 | -86.4 | -12.4 | L | O | Inferior Occipital Gyrus | 18 | OCC |
| 3.13 | 50.8 | -0.6 | -28.2 | R | T | Middle Temporal Gyrus | 21 | TEMP |
| 2.88 | 50.8 | 45.3 | 6.1 | R | F | Middle Frontal Gyrus | 46 | FRONT |
| 2.74 | -18.5 | -8 | -28.9 | L | Limbic | Uncus | 36 | LIMBIC |
| 2.55 | 60.6 | 14.3 | 12.5 | R | F | Inferior Frontal Gyrus | 44 | OBF/IF |
| 2.24 | -28.5 | -53.7 | 59.8 | L | P | Superior PAR Lobule | 7 | PAR |
| 1.64 | 31 | -15.8 | 63.3 | R | F | Precentral Gyrus | 6 | PREM |
| 1.55 | 50.8 | -32.4 | 52.7 | R | P | Inferior PAR Lobule | 40 | PAR |
| 0.99 | -28.5 | 53.4 | 24.8 | L | F | Superior Frontal Gyrus | 10 | OBF/IF |
| 0.96 | -38.5 | 32.4 | 32 | L | F | Middle Frontal Gyrus | 9 | FRONT |
|  |  |  |  |  |  |  |  |  |
|  |  |  |  |  |  |  |  |  |
|  | |  | | |  |  |  |  |
| **SS14**  **MAGN.** | **T-x [mm]** | **T-y [mm]** | **T-z [mm]** | **HEM** | **LOBE** | **GYRUS** | **BA** | **ROI** |
| 5.03 | -58.5 | -56.9 | -2.8 | L | T | Inferior Temporal Gyrus | 37 | FUSIF |
| 4.38 | -28.5 | 46.3 | -2.3 | L | F | Middle Frontal Gyrus | 10 | OBF/IF |
| 4.12 | 1.5 | 57.3 | -9 | R | F | Medial Frontal Gyrus | 10 | OBF/IF |
| 3.84 | -48.5 | -77.2 | -4.2 | L | O | Middle Occipital Gyrus | 19 | OCC |
| 3.30 | -38.5 | -7.8 | 55.2 | L | F | Precentral Gyrus | 6 | PREM |
| 2.96 | -28.5 | -82.1 | 39.5 | L | P | Precuneus | 19 | PAR |
| 2.72 | 21.2 | -1.1 | 65 | R | F | Superior Frontal Gyrus | 6 | PREM |
| 2.63 | 50.8 | 45.3 | 6.1 | R | F | Middle Frontal Gyrus | 46 | FRONT |
| 2.62 | -28.5 | 9.1 | -27.5 | L | T | Superior Temporal Gyrus | 38 | TEMP |
| 2.61 | 11.3 | -96.5 | -13.1 | R | O | Lingual Gyrus | 17 | OCC |
| 2.05 | 50.8 | -61.8 | 41.2 | R | P | Inferior PAR Lobule | 39 | PAR |
| 2.03 | 60.6 | 5.3 | 2.7 | R | T | Superior Temporal Gyrus | 22 | TEMP |
| 2.00 | 60.6 | -2.1 | -13.3 | R | Limbic | Uncus | 20 | LIMBIC |
| 1.97 | 60.6 | -55 | -17.6 | R | O | Fusiform Gyrus | 37 | FUSIF |
|  |  |  |  |  |  |  |  |  |
|  |  |  |  |  |  |  |  |  |
|  | |  | | |  |  |  |  |
| **SS15**  **MAGN.** | **T-x [mm]** | **T-y [mm]** | **T-z [mm]** | **HEM** | **LOBE** | **GYRUS** | **BA** | **ROI** |
| 3.87 | 40.9 | -80.1 | 21.7 | R | T | Middle Temporal Gyrus | 19 | TEMP |
| 3.69 | 40.9 | -88.3 | 3 | R | O | Middle Occipital Gyrus | 18 | OCC |
| 2.35 | -28.5 | -97.5 | -5.7 | L | O | Lingual Gyrus | 18 | OCC |
| 2.16 | 50.8 | 34.3 | 14.2 | R | F | Middle Frontal Gyrus | 46 | FRONT |
| 2.11 | 21.2 | -1.1 | 65 | R | F | Superior Frontal Gyrus | 6 | PREM |
| 1.78 | -18.5 | -1.1 | 65 | L | F | Superior Frontal Gyrus | 6 | PREM |
| 1.56 | -8.5 | -63.8 | 59 | L | P | Superior PAR Lobule | 7 | PAR |
| 1.12 | 50.8 | -42.5 | 51.8 | R | P | Inferior PAR Lobule | 40 | PAR |
| 0.63 | -48.5 | 33.4 | 23.1 | L | F | Middle Frontal Gyrus | 46 | FRONT |

|  | |  | | |  |  |  |  |
| --- | --- | --- | --- | --- | --- | --- | --- | --- |
| **SS16**  **MAGN.** | **T-x [mm]** | **T-y [mm]** | **T-z [mm]** | **HEM** | **LOBE** | **GYRUS** | **BA** | **ROI** |
| 3.97 | 60.6 | -20.3 | 26.8 | R | P | Postcentral Gyrus | 2 | PAR |
| 3.78 | 60.6 | -44.8 | -16.9 | R | T | Inferior Temporal Gyrus | 20 | TEMP |
| 3.72 | -48.5 | -65.1 | -18.4 | L | Cereb | Posterior Lobe, Declive |  | FUSIF |
| 3.65 | -28.5 | -15.8 | 63.3 | L | F | Precentral Gyrus | 6 | PREM |
| 3.53 | 40.9 | -87.3 | -4.9 | R | O | Inferior Occipital Gyrus | 18 | OCC |
| 3.40 | 60.6 | 13.3 | 21.4 | R | F | Inferior Frontal Gyrus | 45 | OBF/IF |
| 3.10 | -48.5 | -0.6 | -28.2 | L | T | Inferior Temporal Gyrus | 20 | TEMP |
| 2.67 | -48.5 | 33.4 | 23.1 | L | F | Middle Frontal Gyrus | 46 | FRONT |
| 2.17 | 21.2 | -16.1 | -22.2 | R | Limbic | Parahippocampal Gyrus | 28 | LIMBIC |
| 2.05 | -8.5 | -63.8 | 59 | L | P | Superior PAR Lobule | 7 | PAR |
|  |  |  |  |  |  |  |  |  |
|  |  |  |  |  |  |  |  |  |
|  | |  | | |  |  |  |  |
| **SS17**  **MAGN.** | **T-x [mm]** | **T-y [mm]** | **T-z [mm]** | **HEM** | **LOBE** | **GYRUS** | **BA** | **ROI** |
| 3.49 | 11.3 | -97.5 | -5.7 | R | O | Lingual Gyrus | 18 | OCC |
| 3.46 | -58.5 | -55 | -17.6 | L | T | Fusiform Gyrus | 37 | FUSIF |
| 3.20 | -18.5 | 52.4 | 33.7 | L | F | Superior Frontal Gyrus | 9 | FRONT |
| 2.69 | -8.5 | -82.1 | 39.5 | L | P | Precuneus | 19 | PAR |
| 1.71 | 31 | -15.8 | 63.3 | R | F | Precentral Gyrus | 6 | PREM |
| 1.47 | 60.6 | -58.9 | 14.5 | R | T | Superior Temporal Gyrus | 22 | TEMP |
| 1.43 | 60.6 | -29.4 | 26 | R | P | Inferior PAR Lobule | 40 | PAR |
| 1.35 | 40.9 | 55.3 | 7 | R | F | Middle Frontal Gyrus | 10 | OBF/IF |
| 1.09 | 11.3 | -9.4 | -14 | R | Limbic | Parahippocampal Gyrus | 34 | LIMBIC |
| 0.98 | -28.5 | -8.5 | 64.2 | L | F | Superior Frontal Gyrus | 6 | OBF/IF |
|  |  |  |  |  |  |  |  |  |
|  |  |  |  |  |  |  |  |  |
|  | |  | | |  |  |  |  |
| **SS18**  **MAGN.** | **T-x [mm]** | **T-y [mm]** | **T-z [mm]** | **HEM** | **LOBE** | **GYRUS** | **BA** | **ROI** |
| 4.93 | 50.8 | 0.4 | 47.2 | R | F | Precentral Gyrus | 6 | PREM |
| 4.28 | 40.9 | -80.1 | 21.7 | R | T | Middle Temporal Gyrus | 19 | TEMP |
| 4.25 | 60.6 | -55.9 | -10.2 | R | T | Inferior Temporal Gyrus | 37 | FUSIF |
| 4.01 | 11.3 | 65.3 | 7.9 | R | F | Superior Frontal Gyrus | 10 | OBF/IF |
| 3.81 | -18.5 | -90.3 | 20.8 | L | O | Cuneus | 18 | OCC |
| 2.27 | -58.5 | -21 | 35.7 | L | P | Postcentral Gyrus | 3 | PAR |
| 1.93 | -58.5 | -2.8 | -5.9 | L | T | Middle temporal Gyrus | 21 | TEMP |
| 1.57 | -18.5 | 30.5 | 49.8 | L | F | Superior Frontal Gyrus | 8 | FRONT |

*Tables of active electromagnetic dipoles in swLORETA for the N400 (400-600 ms) component, obtained in the eighteen participants included in the ANOVA analysis, measured during the “Music” motivational state. MAGN. = magnitude in nA.*

**MOVEMENT**

|  | |  | | |  |  |  |  |
| --- | --- | --- | --- | --- | --- | --- | --- | --- |
| **SS01**  **MAGN.** | **T-x [mm]** | **T-y [mm]** | **T-z [mm]** | **HEM** | **LOBE** | **GYRUS** | **BA** | **ROI** |
| 6.92 | -48.5 | 11.4 | 39.2 | L | F | Middle Frontal Gyrus | 8 | FRONT |
| 6.55 | 50.8 | -68 | 4.7 | R | O | Middle Occipital Gyrus | 37 | FUSIF |
| 6.10 | -58.5 | -5.6 | 28.5 | L | F | Precentral Gyrus | 6 | PREM |
| 4.97 | 50.8 | -0.6 | -28.2 | R | T | Middle Temporal Gyrus | 21 | TEMP |
| 4.44 | -38.5 | 9.1 | -27.5 | L | T | Superior Temporal Gyrus | 38 | TEMP |
| 3.96 | 1.5 | -99.4 | 11 | R | O | Cuneus | 18 | OCC |
| 3.76 | 1.5 | 40.5 | 50.7 | R | F | Superior Frontal Gyrus | 8 | FRONT |
| 3.55 | 60.6 | -6.3 | 37.4 | R | F | Precentral Gyrus | 6 | PREM |
| 3.54 | -58.5 | -55 | -17.6 | L | T | Fusiform Gyrus | 37 | FUSIF |
| 3.03 | -8.5 | -73 | 49.2 | L | P | Precuneus | 7 | PAR |
|  |  |  |  |  |  |  |  |  |
|  |  |  |  |  |  |  |  |  |
|  | |  | | |  |  |  |  |
| **SS02**  **MAGN.** | **T-x [mm]** | **T-y [mm]** | **T-z [mm]** | **HEM** | **LOBE** | **GYRUS** | **BA** | **ROI** |
| 6.36 | 40.9 | -86.4 | -12.4 | R | O | Inferior Occipital Gyrus | 18 | OCC |
| 5.47 | -38.5 | 9.1 | -27.5 | L | T | Superior Temporal Gyrus | 38 | TEMP |
| 5.10 | -48.5 | -33.7 | -23.6 | L | T | Fusiform Gyrus | 20 | FUSIF |
| 4.58 | -48.5 | 36.3 | -3 | L | F | Middle Frontal Gyrus | 47 | OBF/IF |
| 3.89 | 70.5 | -17.5 | -7.3 | R | T | Middle temporal Gyrus | 21 | TEMP |
| 3.81 | -8.5 | -96.5 | -13.1 | L | O | Lingual Gyrus | 17 | OCC |
| 3.28 | 60.6 | 3.3 | 20.5 | R | F | Precentral Gyrus | 6 | PREM |
| 2.97 | -28.5 | 9.5 | 57 | L | F | Middle Frontal Gyrus | 6 | PREM |
| 2.53 | 1.5 | 65.3 | 7.9 | R | F | Medial Frontal Gyrus | 10 | OBF/IF |
|  |  |  |  |  |  |  |  |  |
|  |  |  |  |  |  |  |  |  |
|  | |  | | |  |  |  |  |
| **SS03**  **MAGN.** | **T-x [mm]** | **T-y [mm]** | **T-z [mm]** | **HEM** | **LOBE** | **GYRUS** | **BA** | **ROI** |
| 5.60 | 31 | 56.3 | -1.6 | R | F | Superior Frontal Gyrus | 10 | OBF/IF |
| 3.85 | 60.6 | -55 | -17.6 | R | O | Fusiform Gyrus | 37 | FUSIF |
| 2.99 | -18.5 | -1.1 | 65 | L | F | Superior Frontal Gyrus | 6 | PREM |
| 2.66 | 1.5 | -23.2 | 62.4 | R | F | Medial Frontal Gyrus | 6 | PREM |
| 2.58 | -58.5 | 13.3 | 21.4 | L | F | Inferior Frontal Gyrus | 45 | OBF/IF |
| 2.52 | 11.3 | -63.8 | 59 | R | P | Superior Parietal Lobule | 7 | PAR |
| 2.37 | -58.5 | -55 | -17.6 | L | T | Fusiform Gyrus | 37 | FUSIF |
| 2.18 | 31 | -90.3 | 20.8 | R | O | Middle Occipital Gyrus | 19 | OCC |
| 2.17 | -28.5 | -73 | 49.2 | L | P | Superior Parietal Lobule | 7 | PAR |
| 1.57 | -18.5 | -96.5 | -13.1 | L | O | Lingual Gyrus | 18 | OCC |

|  | |  | | |  |  |  |  |
| --- | --- | --- | --- | --- | --- | --- | --- | --- |
| **SS04**  **MAGN.** | **T-x [mm]** | **T-y [mm]** | **T-z [mm]** | **HEM** | **LOBE** | **GYRUS** | **BA** | **ROI** |
| 4.74 | -58.5 | -44.8 | -16.9 | L | T | Inferior Temporal Gyrus | 20 | TEMP |
| 4.40 | -18.5 | -8 | -28.9 | L | Limbic | Uncus | 36 | LIMBIC |
| 4.12 | -58.5 | 2.4 | 29.4 | L | F | Precentral Gyrus | 6 | PREM |
| 3.72 | 21.2 | 9.1 | -27.5 | R | Limbic | Uncus | 38 | LIMBIC |
| 3.59 | 50.8 | -0.6 | -28.2 | R | T | Middle Temporal Gyrus | 21 | TEMP |
| 2.91 | 21.2 | -55.9 | -10.2 | R | Cereb | Posterior Lobe,Declive |  | FUSIF |
| 2.71 | 60.6 | 13.3 | 21.4 | R | F | Inferior Frontal Gyrus | 45 | OBF/IF |
| 2.02 | 11.3 | -98.5 | 2.1 | R | O | Cuneus | 17 | OCC |
| 1.27 | -18.5 | -53.7 | 59.8 | L | P | Superior Parietal Lobule | 7 | PAR |
| 1.22 | 60.6 | -41.5 | 42.9 | R | P | Inferior Parietal Lobule | 40 | PAR |

|  | |  | | |  |  |  |  |
| --- | --- | --- | --- | --- | --- | --- | --- | --- |
| **SS05**  **MAGN.** | **T-x [mm]** | **T-y [mm]** | **T-z [mm]** | **HEM** | **LOBE** | **GYRUS** | **BA** | **ROI** |
| 3.69 | -38.5 | -81.1 | 30.6 | L | O | Superior Occipital Gyrus | 19 | OCC |
| 2.95 | -58.5 | -5.6 | 28.5 | L | F | Precentral Gyrus | 6 | PREM |
| 2.28 | -68.5 | -36.6 | -1.3 | L | T | Middle Temporal Gyrus | 21 | TEMP |
| 2.26 | 50.8 | 1.4 | 38.3 | R | F | Middle Frontal Gyrus | 6 | PREM |
| 2.20 | 31 | -97.5 | -5.7 | R | O | Inferior Occipital Gyrus | 18 | OCC |
| 1.57 | -18.5 | 52.4 | 33.7 | L | F | Superior Frontal Gyrus | 9 | FRONT |
| 1.53 | 60.6 | -55.9 | -10.2 | R | T | Inferior Temporal Gyrus | 37 | FUSIF |
| 1.31 | 70.5 | -36.6 | -1.3 | R | T | Middle Temporal Gyrus | 21 | TEMP |
|  |  |  |  |  |  |  |  |  |
|  |  |  |  |  |  |  |  |  |
|  | |  | | |  |  |  |  |
| **SS06**  **MAGN.** | **T-x [mm]** | **T-y [mm]** | **T-z [mm]** | **HEM** | **LOBE** | **GYRUS** | **BA** | **ROI** |
| 4.60 | -38.5 | -62.8 | 50.1 | L | P | Superior Parietal Lobule | 7 | PAR |
| 4.08 | -48.5 | -76.2 | -11.7 | L | T | Fusiform Gyrus | 19 | FUSIF |
| 3.56 | 50.8 | -0.6 | -28.2 | R | T | Middle Temporal Gyrus | 21 | TEMP |
| 3.47 | 60.6 | -55 | -17.6 | R | O | Fusiform Gyrus | 37 | FUSIF |
| 3.32 | -38.5 | -8 | -28.9 | L | T | Middle Temporal Gyrus | 21 | TEMP |
| 3.05 | 1.5 | -99.4 | 11 | R | O | Cuneus | 18 | OCC |
| 2.94 | -48.5 | 22.4 | 31.1 | L | F | Middle Frontal Gyrus | 9 | FRONT |
| 2.91 | -48.5 | -7 | 46.3 | L | F | Precentral Gyrus | 4 | PREM |
| 1.93 | 21.2 | -1.1 | 65 | R | F | Superior Frontal Gyrus | 6 | PREM |
| 1.90 | 21.2 | 52.4 | 33.7 | R | F | Superior Frontal Gyrus | 9 | FRONT |
| 1.81 | 1.5 | 65.3 | 7.9 | R | F | Medial Frontal Gyrus | 10 | OBF/IF |
| 1.62 | -8.5 | 57.3 | -9 | L | F | Superior Frontal Gyrus | 10 | OBF/IF |

|  | |  | | |  |  |  |  |
| --- | --- | --- | --- | --- | --- | --- | --- | --- |
| **SS07**  **MAGN.** | **T-x [mm]** | **T-y [mm]** | **T-z [mm]** | **HEM** | **LOBE** | **GYRUS** | **BA** | **ROI** |
| 3.99 | -58.5 | -1.4 | -20.8 | L | T | Middle temporal Gyrus | 21 | TEMP |
| 3.43 | -58.5 | 4.3 | 11.6 | L | F | Precentral Gyrus | 6 | PREM |
| 2.83 | -48.5 | -33.7 | -23.6 | L | T | Fusiform Gyrus | 20 | FUSIF |
| 2.69 | -48.5 | 33.4 | 23.1 | L | F | Middle Frontal Gyrus | 46 | FRONT |
| 2.64 | 50.8 | -16.1 | -22.2 | R | T | Fusiform Gyrus | 20 | FUSIF |
| 2.27 | 31 | -15.3 | -29.6 | R | Limbic | Uncus | 20 | LIMBIC |
| 1.90 | 40.9 | -88.3 | 3 | R | O | Middle Occipital Gyrus | 18 | OCC |
| 1.84 | 50.8 | -32.4 | 52.7 | R | P | Inferior Parietal Lobule | 40 | PAR |
| 1.78 | 60.6 | -6.3 | 37.4 | R | F | Precentral Gyrus | 6 | PREM |
| 1.32 | 1.5 | -63.8 | 59 | R | P | Precuneus | 7 | PAR |

|  | |  | | |  |  |  |  |
| --- | --- | --- | --- | --- | --- | --- | --- | --- |
| **SS08**  **MAGN.** | **T-x [mm]** | **T-y [mm]** | **T-z [mm]** | **HEM** | **LOBE** | **GYRUS** | **BA** | **ROI** |
| 2.92 | 40.9 | -81.1 | 30.6 | R | O | Superior Occipital Gyrus | 19 | OCC |
| 2.60 | 31 | -53.7 | 59.8 | R | P | Superior Parietal Lobule | 7 | PAR |
| 2.54 | 60.6 | -55 | -17.6 | R | O | Fusiform Gyrus | 37 | FUSIF |
| 2.40 | 50.8 | -0.6 | -28.2 | R | T | Middle Temporal Gyrus | 21 | TEMP |
| 2.13 | -38.5 | -86.4 | -12.4 | L | O | Inferior Occipital Gyrus | 18 | OCC |
| 1.97 | 31 | -15.3 | -29.6 | R | Limbic | Uncus | 20 | LIMBIC |
| 1.63 | -8.5 | -0.6 | -28.2 | L | Limbic | Uncus | 28 | LIMBIC |
| 1.43 | -58.5 | -8.7 | -21.5 | L | T | Inferior Temporal Gyrus | 20 | TEMP |
| 1.28 | -38.5 | 32.4 | 32 | L | F | Middle Frontal Gyrus | 9 | FRONT |
| 1.25 | 50.8 | 33.4 | 23.1 | R | F | Middle Frontal Gyrus | 46 | FRONT |
| 0.68 | -58.5 | -31.4 | 43.8 | L | P | Inferior Parietal Lobule | 40 | PAR |
|  |  |  |  |  |  |  |  |  |
|  | |  | | |  |  |  |  |
| **SS09**  **MAGN.** | **T-x [mm]** | **T-y [mm]** | **T-z [mm]** | **HEM** | **LOBE** | **GYRUS** | **BA** | **ROI** |
| 4.13 | -68.5 | -36.6 | -1.3 | L | T | Middle Temporal Gyrus | 21 | TEMP |
| 4.05 | -58.5 | -55 | -17.6 | L | T | Fusiform Gyrus | 37 | FUSIF |
| 3.93 | -38.5 | -0.4 | 56.1 | L | F | Middle Frontal Gyrus | 6 | PREM |
| 3.18 | -28.5 | -82.1 | 39.5 | L | P | Precuneus | 19 | PAR |
| 3.07 | -48.5 | 35.3 | 5.3 | L | F | Inferior Frontal Gyrus | 45 | OBF/IF |
| 2.89 | 50.8 | -16.1 | -22.2 | R | T | Fusiform Gyrus | 20 | FUSIF |
| 2.66 | -28.5 | -97.5 | -5.7 | L | O | Lingual Gyrus | 18 | OCC |
| 2.46 | 31 | -15.3 | -29.6 | R | Limbic | Uncus | 20 | LIMBIC |
| 1.73 | 21.2 | -1.1 | 65 | R | F | Superior Frontal Gyrus | 6 | PREM |
| 1.64 | 40.9 | -88.3 | 3 | R | O | Middle Occipital Gyrus | 18 | OCC |
| 1.51 | 60.6 | 14.3 | 12.5 | R | F | Inferior Frontal Gyrus | 44 | OBF/IF |
| 1.15 | 60.6 | -41.5 | 42.9 | R | P | Inferior Parietal Lobule | 40 | PAR |

|  | |  | | |  |  |  |  |
| --- | --- | --- | --- | --- | --- | --- | --- | --- |
| **SS10**  **MAGN.** | **T-x [mm]** | **T-y [mm]** | **T-z [mm]** | **HEM** | **LOBE** | **GYRUS** | **BA** | **ROI** |
| 2.78 | 31 | -90.3 | 20.8 | R | O | Middle Occipital Gyrus | 19 | OCC |
| 2.34 | -58.5 | -8.7 | -21.5 | L | T | Inferior Temporal Gyrus | 20 | TEMP |
| 1.90 | -18.5 | -96.5 | -13.1 | L | O | Lingual Gyrus | 18 | OCC |
| 1.80 | -28.5 | -15.8 | 63.3 | L | F | Precentral Gyrus | 6 | PREM |
| 1.50 | 70.5 | -17.5 | -7.3 | R | T | Middle temporal Gyrus | 21 | TEMP |
| 1.38 | 31 | -55.9 | -10.2 | R | O | Fusiform Gyrus, WM |  | FUSIF |
| 1.38 | -8.5 | -63.8 | 59 | L | Limbic | Parahippocampal Gyrus | 19 | LIMBIC |
| 1.19 | 21.2 | -1.1 | 65 | R | F | Superior Frontal Gyrus | 6 | PREM |
| 1.14 | -28.5 | 53.4 | 24.8 | L | F | Superior Frontal Gyrus | 10 | OBF/IF |
| 1.00 | 50.8 | -32.4 | 52.7 | R | P | Inferior Parietal Lobule | 40 | PAR |
|  |  |  |  |  |  |  |  |  |
|  |  |  |  |  |  |  |  |  |
|  | |  | | |  |  |  |  |
| **SS11**  **MAGN.** | **T-x [mm]** | **T-y [mm]** | **T-z [mm]** | **HEM** | **LOBE** | **GYRUS** | **BA** | **ROI** |
| 3.42 | -28.5 | 53.4 | 24.8 | L | F | Superior Frontal Gyrus | 10 | OBF/IF |
| 2.61 | 1.5 | 48.2 | -17.2 | R | F | Medial Frontal Gyrus | 11 | OBF/IF |
| 2.55 | 60.6 | -29.4 | 26 | R | P | Inferior Parietal Lobule | 40 | PAR |
| 2.41 | 40.9 | -86.4 | -12.4 | R | O | Inferior Occipital Gyrus | 18 | OCC |
| 2.28 | -28.5 | -23.2 | 62.4 | L | F | Precentral Gyrus | 4 | PREM |
| 2.27 | 50.8 | -70 | 22.5 | R | T | Middle Temporal Gyrus | 39 | TEMP |
| 2.05 | 60.6 | -55 | -17.6 | R | O | Fusiform Gyrus | 37 | FUSIF |
| 1.67 | 1.5 | -33.4 | 61.6 | R | F | Paracentral Lobule | 6 | PREM |
| 1.61 | -8.5 | -63.8 | 59 | L | P | Superior Parietal Lobule | 7 | PAR |
| 1.54 | -58.5 | -55 | -17.6 | L | T | Fusiform Gyrus | 37 | FUSIF |
| 1.25 | 1.5 | -29.4 | 26 | R | Limbic | Cingulate Gyrus | 23 | LIMBIC |

|  | |  | | |  |  |  |  |
| --- | --- | --- | --- | --- | --- | --- | --- | --- |
| **SS12**  **MAGN.** | **T-x [mm]** | **T-y [mm]** | **T-z [mm]** | **HEM** | **LOBE** | **GYRUS** | **BA** | **ROI** |
| 5.15 | 31 | 56.3 | -1.6 | R | F | Superior Frontal Gyrus | 10 | OBF/IF |
| 4.39 | -58.5 | -55 | -17.6 | L | T | Fusiform gyrus | 37 | FUSIF |
| 3.29 | 21.2 | -82.1 | 39.5 | R | P | Precuneus | 19 | PAR |
| 3.19 | 60.6 | -55 | -17.6 | R | O | Fusiform gyrus | 37 | FUSIF |
| 3.14 | 21.2 | -15.8 | 63.3 | R | F | Precentral Gyrus | 6 | PREM |
| 2.89 | -18.5 | 41.4 | 41.8 | L | F | Superior Frontal Gyrus | 8 | FRONT |
| 2.70 | 50.8 | -0.6 | -28.2 | R | T | Middle Temporal Gyrus | 21 | TEMP |
| 2.45 | -8.5 | -99.4 | 11 | L | O | Cuneus | 18 | OCC |
| 2.41 | -18.5 | -23.2 | 62.4 | L | F | Precentral Gyrus | 4 | PREM |
| 2.05 | -8.5 | -0.6 | -28.2 | L | Limbic | Uncus | 28 | LIMBIC |
| 2.01 | -58.5 | -31.4 | 43.8 | L | P | Inferior Parietal Lobule | 40 | PAR |

|  | |  | | |  |  |  |  |
| --- | --- | --- | --- | --- | --- | --- | --- | --- |
| **SS13**  **MAGN.** | **T-x [mm]** | **T-y [mm]** | **T-z [mm]** | **HEM** | **LOBE** | **GYRUS** | **BA** | **ROI** |
| 4.30 | 60.6 | -55.9 | -10.2 | R | T | Inferior Temporal Gyrus | 37 | FUSIF |
| 3.06 | 60.6 | -41.5 | 42.9 | R | P | Inferior Parietal Lobule | 40 | PAR |
| 2.96 | 21.2 | 30.5 | 49.8 | R | F | Superior Frontal Gyrus | 8 | FRONT |
| 2.66 | -58.5 | -55 | -17.6 | L | T | Fusiform Gyrus | 37 | FUSIF |
| 2.65 | -28.5 | 9.1 | -27.5 | L | T | Superior Temporal Gyrus | 38 | TEMP |
| 2.41 | 50.8 | 17.2 | -11.9 | R | T | Superior Temporal Gyrus | 38 | TEMP |
| 2.34 | 60.6 | -2.1 | -13.3 | R | Limbic | Uncus | 20 | LIMBIC |
| 2.19 | 11.3 | -99.4 | 11 | R | O | Cuneus | 18 | OCC |
| 2.18 | 31 | -23.2 | 62.4 | R | F | Precentral Gyrus | 4 | PREM |
| 1.10 | -18.5 | -23.2 | 62.4 | L | F | Precentral Gyrus | 4 | PREM |
| 0.99 | -8.5 | 64.4 | 16.8 | L | F | Superior Frontal Gyrus | 10 | OBF/IF |
|  |  |  |  |  |  |  |  |  |
|  |  |  |  |  |  |  |  |  |
|  | |  | | |  |  |  |  |
| **SS14**  **MAGN.** | **T-x [mm]** | **T-y [mm]** | **T-z [mm]** | **HEM** | **LOBE** | **GYRUS** | **BA** | **ROI** |
| 7.97 | -58.5 | -1.4 | -20.8 | L | T | Middle temporal Gyrus | 21 | TEMP |
| 7.89 | 60.6 | -55.9 | -10.2 | R | T | Inferior Temporal Gyrus | 37 | FUSIF |
| 7.48 | 70.5 | -36.6 | -1.3 | R | T | Middle Temporal Gyrus | 21 | TEMP |
| 6.28 | -58.5 | -55 | -17.6 | L | T | Fusiform Gyrus | 37 | FUSIF |
| 5.84 | 1.5 | 65.3 | 7.9 | R | F | Medial Frontal Gyrus | 10 | OBF/IF |
| 4.81 | -8.5 | -99.4 | 11 | L | O | Cuneus | 18 | OCC |
| 4.78 | 21.2 | -98.5 | 2.1 | R | O | Cuneus | 18 | OCC |
| 4.38 | -38.5 | -62.8 | 50.1 | L | P | Superior Parietal Lobule | 7 | PAR |
| 4.24 | 40.9 | -62.8 | 50.1 | R | P | Superior Parietal Lobule | 7 | PAR |
| 3.03 | 21.2 | 9.1 | -27.5 | R | Limbic | Uncus | 38 | LIMBIC |
| 2.70 | 50.8 | 22.4 | 31.1 | R | F | Middle Frontal Gyrus | 9 | FRONT |
| 2.67 | 60.6 | -6.3 | 37.4 | R | F | Precentral Gyrus | 6 | PREM |

|  | |  | | |  |  |  |  |
| --- | --- | --- | --- | --- | --- | --- | --- | --- |
| **SS15**  **MAGN.** | **T-x [mm]** | **T-y [mm]** | **T-z [mm]** | **HEM** | **LOBE** | **GYRUS** | **BA** | **ROI** |
| 6.32 | -8.5 | 65.3 | 7.9 | L | F | Superior Frontal Gyrus | 10 | OBF/IF |
| 5.86 | 1.5 | 57.3 | -9 | R | F | Medial Frontal Gyrus | 10 | OBF/IF |
| 1.40 | 40.9 | -55 | -17.6 | R | O | Fusiform Gyrus | 37 | FUSIF |
| 1.26 | -38.5 | -75.2 | -19.1 | L | Cereb | Posterior Lobe,Declive |  | FUSIF |
| 1.17 | 21.2 | -15.8 | 63.3 | R | F | Precentral Gyrus | 6 | PREM |
| 0.75 | -58.5 | -31.4 | 43.8 | L | P | Inferior Parietal Lobule | 40 | PAR |

|  | |  | | |  |  |  |  |
| --- | --- | --- | --- | --- | --- | --- | --- | --- |
| **SS16**  **MAGN.** | **T-x [mm]** | **T-y [mm]** | **T-z [mm]** | **HEM** | **LOBE** | **GYRUS** | **BA** | **ROI** |
| 3.44 | 31 | -15.8 | 63.3 | R | F | Precentral Gyrus | 6 | PREM |
| 3.07 | 50.8 | 33.4 | 23.1 | R | F | Middle Frontal Gyrus | 46 | FRONT |
| 2.10 | -58.5 | -58.9 | 14.5 | L | T | Superior Temporal Gyrus | 22 | TEMP |
| 1.71 | -48.5 | -71 | 31.4 | L | P | Angular Gyrus | 39 | PAR |
| 1.40 | 50.8 | -71 | 31.4 | R | T | Angular Gyru | 39 | TEMP |
| 1.21 | 40.9 | -86.4 | -12.4 | R | O | Inferior Occipital Gyrus | 18 | OCC |
| 1.20 | 60.6 | -55.9 | -10.2 | R | T | Inferior Temporal Gyrus | 37 | FUSIF |
| 1.19 | 60.6 | -39.6 | 25.1 | R | P | Inferior Parietal Lobule | 40 | PAR |
| 1.04 | 1.5 | 48.2 | -17.2 | R | F | Medial Frontal Gyrus | 11 | OBF/IF |
| 0.97 | -38.5 | 46.3 | -2.3 | L | F | Inferior Frontal Gyrus | 10 | OBF/IF |
| 0.85 | -8.5 | -0.6 | -28.2 | L | Limbic | Uncus | 28 | LIMBIC |

|  | |  | | |  |  |  |  |
| --- | --- | --- | --- | --- | --- | --- | --- | --- |
| **SS17**  **MAGN.** | **T-x [mm]** | **T-y [mm]** | **T-z [mm]** | **HEM** | **LOBE** | **GYRUS** | **BA** | **ROI** |
| 5.24 | 21.2 | -97.5 | -5.7 | R | O | Lingual Gyrus | 18 | OCC |
| 4.14 | -58.5 | -55 | -17.6 | L | T | Fusiform Gyrus | 37 | FUSIF |
| 2.84 | 60.6 | -6.3 | 37.4 | R | F | Precentral Gyrus | 6 | PREM |
| 2.33 | -48.5 | 34.3 | 14.2 | L | F | Middle Frontal Gyrus | 46 | FRONT |
| 2.06 | 60.6 | -24.5 | -15.5 | R | T | Inferior Temporal Gyrus | 20 | TEMP |
| 1.95 | -8.5 | -8.5 | 64.2 | L | F | Medial Frontal Gyrus | 6 | PREM |
| 0.94 | 11.3 | 65.3 | 7.9 | R | F | Superior Frontal Gyrus | 10 | OBF/IF |
|  |  |  |  |  |  |  |  |  |
|  |  |  |  |  |  |  |  |  |
|  | |  | | |  |  |  |  |
| **SS18**  **MAGN.** | **T-x [mm]** | **T-y [mm]** | **T-z [mm]** | **HEM** | **LOBE** | **GYRUS** | **BA** | **ROI** |
| 3.69 | 60.6 | -21 | 35.7 | R | P | Postcentral Gyrus | 3 | PAR |
| 2.62 | 70.5 | -17.5 | -7.3 | R | T | Middle temporal Gyrus | 21 | TEMP |
| 2.52 | 11.3 | 65.3 | 7.9 | R | F | Superior Frontal Gyrus | 10 | OBF/IF |
| 2.51 | -58.5 | -2.8 | -5.9 | L | T | Middle temporal Gyrus | 21 | TEMP |
| 2.39 | 40.9 | -81.1 | 30.6 | R | O | Superior Occipital Gyrus | 19 | OCC |
| 2.39 | -18.5 | -90.3 | 20.8 | L | O | Cuneus | 18 | OCC |
| 2.23 | 50.8 | 33.4 | 23.1 | R | F | Middle Frontal Gyrus | 46 | FRONT |
| 1.50 | 60.6 | -55.9 | -10.2 | R | T | Inferior Temporal Gyrus | 37 | FUSIF |
| 1.11 | -18.5 | -63.8 | 59 | L | P | Superior Parietal Lobule | 7 | PAR |

*Tables of active electromagnetic dipoles in swLORETA for the N400 (400-600 ms) component, obtained in the eighteen participants included in the ANOVA analysis, measured during the “Movement” motivational state. MAGN. = magnitude in nA).*
